# Intra-host evolution during SARS-CoV-2 prolonged infection

**DOI:** 10.1093/ve/veab078

**Published:** 2021-09-29

**Authors:** Carolina M Voloch, Ronaldo da Silva Francisco Jr, Luiz G P de Almeida, Otavio J Brustolini, Cynthia C Cardoso, Alexandra L Gerber, Ana Paula de C Guimarães, Isabela de Carvalho Leitão, Diana Mariani, Victor Akira Ota, Cristiano X Lima, Mauro M Teixeira, Ana Carolina F Dias, Rafael Mello Galliez, Débora Souza Faffe, Luís Cristóvão Pôrto, Renato S Aguiar, Terezinha M P P Castiñeira, Orlando C Ferreira, Amilcar Tanuri, Ana Tereza R de Vasconcelos

**Affiliations:** Departamento de Genética, Instituto de Biologia, Universidade Federal do Rio de Janeiro, Av. Carlos Chagas Filho, 373 - Cidade Universitária da Universidade Federal do Rio de Janeiro - Ilha do Fundão, Rio de Janeiro 21941-902, Brazil; Laboratório de Bioinformática, Laboratório Nacional de Computação Científica, Av. Getúlio Vargas, 333 - Quitandinha, Petrópolis 25651-076, Brazil; Laboratório de Bioinformática, Laboratório Nacional de Computação Científica, Av. Getúlio Vargas, 333 - Quitandinha, Petrópolis 25651-076, Brazil; Laboratório de Bioinformática, Laboratório Nacional de Computação Científica, Av. Getúlio Vargas, 333 - Quitandinha, Petrópolis 25651-076, Brazil; Departamento de Genética, Instituto de Biologia, Universidade Federal do Rio de Janeiro, Av. Carlos Chagas Filho, 373 - Cidade Universitária da Universidade Federal do Rio de Janeiro - Ilha do Fundão, Rio de Janeiro 21941-902, Brazil; Laboratório de Bioinformática, Laboratório Nacional de Computação Científica, Av. Getúlio Vargas, 333 - Quitandinha, Petrópolis 25651-076, Brazil; Laboratório de Bioinformática, Laboratório Nacional de Computação Científica, Av. Getúlio Vargas, 333 - Quitandinha, Petrópolis 25651-076, Brazil; Instituto de Biofísica, Universidade Federal do Rio de Janeiro, Av. Carlos Chagas Filho, 373 - Cidade Universitária da Universidade Federal do Rio de Janeiro - Ilha do Fundão, Rio de Janeiro 21941-170, Brazil; Departamento de Genética, Instituto de Biologia, Universidade Federal do Rio de Janeiro, Av. Carlos Chagas Filho, 373 - Cidade Universitária da Universidade Federal do Rio de Janeiro - Ilha do Fundão, Rio de Janeiro 21941-902, Brazil; Departamento de Doenças Infecciosas e Parasitárias, Faculdade de Medicina, Universidade Federal do Rio de Janeiro, Av. Carlos Chagas Filho, 373, Edifício do Centro de Ciências da Saúde, Cidade Universitária da Universidade Federal do Rio de Janeiro - Ilha do Fundão, Rio de Janeiro 21941-902, Brazil; Departamento de Cirurgia, Faculdade de Medicina, Universidade Federal de Minas Gerais, Av. Prof. Alfredo Balena, 190 - Santa Efigênia, Belo Horizonte, MG 30130-100, Brazil; Simile Instituto de Imunologia Aplicada Ltda. R. São Paulo, 1932, Belo Horizonte, 30170-132, Brazil; Departamento de Bioquimica e Imunologia, Universidade Federal de Minas Gerais, Av. Antônio Carlos, 6627 - Pampulha, Belo Horizonte 31270-901, Brazil; Simile Instituto de Imunologia Aplicada Ltda. R. São Paulo, 1932, Belo Horizonte, 30170-132, Brazil; Departamento de Bioquimica e Imunologia, Universidade Federal de Minas Gerais, Av. Antônio Carlos, 6627 - Pampulha, Belo Horizonte 31270-901, Brazil; Departamento de Doenças Infecciosas e Parasitárias, Faculdade de Medicina, Universidade Federal do Rio de Janeiro, Av. Carlos Chagas Filho, 373, Edifício do Centro de Ciências da Saúde, Cidade Universitária da Universidade Federal do Rio de Janeiro - Ilha do Fundão, Rio de Janeiro 21941-902, Brazil; Instituto de Biofísica, Universidade Federal do Rio de Janeiro, Av. Carlos Chagas Filho, 373 - Cidade Universitária da Universidade Federal do Rio de Janeiro - Ilha do Fundão, Rio de Janeiro 21941-170, Brazil; Instituto de Biologia Roberto Alcântara Gomes, Universidade do Estado do Rio de Janeiro, Boulevard 28 de Setembro, 87, Rio de Janeiro 20511-010, Brazil; Instituto de Biofísica, Universidade Federal do Rio de Janeiro, Av. Carlos Chagas Filho, 373 - Cidade Universitária da Universidade Federal do Rio de Janeiro - Ilha do Fundão, Rio de Janeiro 21941-170, Brazil; Departamento de Genética, Instituto de Biologia, Universidade Federal do Rio de Janeiro, Av. Carlos Chagas Filho, 373 - Cidade Universitária da Universidade Federal do Rio de Janeiro - Ilha do Fundão, Rio de Janeiro 21941-902, Brazil; Departamento de Genética, Ecologia e Evolução, Instituto de Ciências Biológicas, Universidade Federal de Minas Gerais, Av. Antônio Carlos, 6627, Belo Horizonte 31270-901, Brazil; Instituto D’Or de Pesquisa e Ensino (IDOR), Rua Diniz Cordeiro, 30, Rio de Janeiro 22281-100, Brazil; Departamento de Doenças Infecciosas e Parasitárias, Faculdade de Medicina, Universidade Federal do Rio de Janeiro, Av. Carlos Chagas Filho, 373, Edifício do Centro de Ciências da Saúde, Cidade Universitária da Universidade Federal do Rio de Janeiro - Ilha do Fundão, Rio de Janeiro 21941-902, Brazil; Departamento de Genética, Instituto de Biologia, Universidade Federal do Rio de Janeiro, Av. Carlos Chagas Filho, 373 - Cidade Universitária da Universidade Federal do Rio de Janeiro - Ilha do Fundão, Rio de Janeiro 21941-902, Brazil; Departamento de Genética, Instituto de Biologia, Universidade Federal do Rio de Janeiro, Av. Carlos Chagas Filho, 373 - Cidade Universitária da Universidade Federal do Rio de Janeiro - Ilha do Fundão, Rio de Janeiro 21941-902, Brazil; Laboratório de Bioinformática, Laboratório Nacional de Computação Científica, Av. Getúlio Vargas, 333 - Quitandinha, Petrópolis 25651-076, Brazil

**Keywords:** COVID-19, RNA-editing enzymes, prolonged infection, Spike gene, helicase gene

## Abstract

Long-term infection of severe acute respiratory syndrome coronavirus 2 (SARS-CoV-2) represents a challenge to virus dispersion and the control of coronavirus disease 2019 (COVID-19) pandemic. The reason why some people have prolonged infection and how the virus persists for so long are still not fully understood. Recent studies suggested that the accumulation of intra-host single nucleotide variants (iSNVs) over the course of the infection might play an important role in persistence as well as emergence of mutations of concern. For this reason, we aimed to investigate the intra-host evolution of SARS-CoV-2 during prolonged infection. Thirty-three patients who remained reverse transcription polymerase chain reaction (RT-PCR) positive in the nasopharynx for on average 18 days from the symptoms onset were included in this study. Whole-genome sequences were obtained for each patient at two different time points. Phylogenetic, populational, and computational analyses of viral sequences were consistent with prolonged infection without evidence of coinfection in our cohort. We observed an elevated within-host genomic diversity at the second time point samples positively correlated with cycle threshold (Ct) values (lower viral load). Direct transmission was also confirmed in a small cluster of healthcare professionals that shared the same workplace by the presence of common iSNVs. A differential accumulation of missense variants between the time points was detected targeting crucial structural and non-structural proteins such as Spike and helicase. Interestingly, longitudinal acquisition of iSNVs in Spike protein coincided in many cases with SARS-CoV-2 reactive and predicted T cell epitopes. We observed a distinguishing pattern of mutations over the course of the infection mainly driven by increasing A→U and decreasing G→A signatures. G→A mutations may be associated with RNA-editing enzyme activities; therefore, the mutational profiles observed in our analysis were suggestive of innate immune mechanisms of the host cell defense. Therefore, we unveiled a dynamic and complex landscape of host and pathogen interaction during prolonged infection of SARS-CoV-2, suggesting that the host’s innate immunity shapes the increase of intra-host diversity. Our findings may also shed light on possible mechanisms underlying the emergence and spread of new variants resistant to the host immune response as recently observed in COVID-19 pandemic.

## 1. Introduction

Prolonged severe acute respiratory syndrome coronavirus 2 (SARS-CoV-2) infection represents a great challenge to the development of effective public health policies to control the coronavirus disease 2019 (COVID-19) pandemic. The average time between symptoms onset and the first negative reverse transcription polymerase chain reaction (RT-PCR) test has been described as 15–17 days in nasopharynx, although longer periods are often observed, varying according to the clinical specimen (Pavon et al., 2020; Sun et al., 2020; Xu et al., 2020). Despite this fact, due to the low availability of PCR tests, there is a prevalent use of symptom-based criteria for interruption of in-home isolation (Centers for Disease Control and Prevention; CDC 2021).

Persistent infection has already been described for many other respiratory viruses, including influenza (Wang et al., 2018), Middle East respiratory syndrome (Arabi et al., 2018), and respiratory syncytial virus (Gomez 2012). The reasons why some people have long-term infection are still not fully understood. Even though this phenomenon has been associated with immunocompromised patients (O’Sullivan et al., 2020; Camprubí et al., 2020; Abdul-Jawad et al., 2021; Baang et al., 2021; Hensley et al., 2021; Siqueira et al., 2021), about 10–30 per cent of COVID-19 patients worldwide may experience symptoms for 3–12 weeks (Greenhalgh et al., 2020; Ladds et al., 2020). The increasing number of prolonged COVID-19 cases indicates that this may not be a rare phenomenon and needs to be explored to better control epidemic spread.

SARS-CoV-2 prolonged infection is characterized mainly by continued PCR positivity while, overall, SARS-CoV-2 viral load is reduced after 10 days of infection (Byrne et al., 2020). Nevertheless, the period of infection may be affected by several factors, including viral load, disease severity and, as above-mentioned, immunological status of the patients (Bullard et al., 2020; Baang et al., 2021; Camprubí et al., 2020; Han et al., 2021; Kampen et al., 2021; Adrielle Dos Santos et al., 2021). In addition to the uncontrolled transmission, prolonged infection in immunocompromised patients was suggested to have shaped the emergence of the variants of concern observed around the world (Avanzato et al., 2020; Choi et al., 2020; Kemp et al., 2020). Finally, intra-host SARS-CoV-2 diversity and variations in viral populations along the disease course have already been characterized (Wang et al., 2021a,b; Lythgoe et al., 2021; Valesano et al., 2021; Jary et al., 2020; Karamitros et al., 2020).

These findings raise questions about the underlying mechanism and also possible consequences of prolonged infection of SARS-CoV-2. Here, we investigated a series of 33 patients from the cities of Rio de Janeiro and Belo Horizonte (Brazil), who remained RT-PCR positive for on average 18 days from the symptoms until the last positive test. Samples were obtained at two different time points for each patient. Phylogenetic, populational, and computational analyses of SARS-CoV-2 sequences confirmed prolonged infections and showed increasing diversity associated with persistence, immune escape-related mutations, and editing signatures of APOBEC-induced interferon enzymes.

## 2. Materials and methods

### 2.1 Study participants and sample collection

Thirty-three individuals with prolonged infection by SARS-CoV-2 were enrolled in the study. Subjects from both genders were recruited at the Center for COVID-19 diagnosis from the Federal University of Rio de Janeiro and Simile Medicina Diagnóstica at Belo Horizonte from March to June 2020. Prolonged cases were defined as those who remained positive for SARS-CoV-2 RNA in nasopharyngeal samples for at least 14 days since the onset of symptoms. We defined infection time as the interval between the symptoms onset and the last positive RT-PCR test. Detection of SARS-CoV-2 and human RNase P RNA were performed by RT-PCR using the CDC protocol (Waggoner et al., 2020). Blood samples and nasopharyngeal swabs were obtained from each patient at two time points, and time 1 (T1) was determined as the first sample with a positive RT-PCR test. Serology tests were also performed for all samples (see Supplementary material). Clinical and demographic data were self-reported by the patients. The present study was approved by the National Commission of Ethics in Research (protocol numbers 30161620.0.0000.5257 and 30127020.0.0000.0068). Written informed consent was obtained from all participants.

### 2.2 Next-generation sequencing and data analysis

Total RNA from SARS-CoV-2-positive samples was converted to cDNA using the SuperScript IV First-Strand Synthesis System (Thermo Fisher Scientific, USA). Viral whole-genome amplification was performed according to the Artic Network protocol (https://artic.network/ncov-2019) using the SARS-CoV-2 primer scheme (V3). Sequencing libraries were constructed with the TruSeq DNA Nano kit (Illumina, USA) as described by the manufacturer. Libraries were sequenced in a MiSeq System with MiSeq Reagent Kit v3 (Illumina, USA) set to obtain 2 × 250 bp reads.

Next, raw read sequences in FASTQ format were first pre-processed using FastQC (v0.11.4) (https://www.bioinformatics.babraham.ac.uk/projects/fastqc/) and trimmomatic v0.39 (Bolger, Lohse, and Usadel 2014) for quality control and low-quality reads filtration, keeping those with an average quality ≥ 25. Bioinformatic pipeline for next-generation sequencing (NGS) data analysis include removing optical duplicates with cutadapt v2.1 (Martin 2011) and clumpify v38.41 (https://sourceforge.net/projects/bbmap/); read mapping to the reference genome (NC_045512.2) using the BWA 0.7.17 (Martin 2011; Li and Durbin 2009); and post-processing steps with samtools v1.10 (Li et al., 2009) and picard v2.17.0 packages (http://broadinstitute.github.io/picard/).

We also performed *de novo* assembly using megahit programs v.1.1.4 (Li et al., 2015) and skesa v2.4.0 (Souvorov, Agarwala, and Lipman 2018). Single-nucleotide variants (SNVs) were detected using variant calling protocol from GATK v4.1.7.0 (DePristo et al., 2011) and LoFreq v 2.1.5 (Wilm et al., 2012) for high- and low-frequency SNVs, respectively. We further generated a consensus genome sequence from high-frequency SNVs for each sample using bcftools v1.10.2 and bedtools v2.29.2 (Li 2011a,b; Quinlan and Hall 2010). The GATK and LoFreq results were combined, and a pairwise variant filtration analysis was performed using the following criteria: (1) average base quality criteria ≥ 15; (2) allele frequency ≥ 5 per cent, and (3) minimum coverage ≥ 100 in both samples of the pair for non-lineage defining mutations. All variants were annotated using snpEff v4.5 (Cingolani et al., 2012).

To better characterize the within-host viral diversity, possible coinfection events underlying prolonged infection, as well as population structure and dynamics, a machine learning model based on the Random Forest algorithm with Repeated Cross Validation of 100.000 repetitions was applied using caret R package (Kuhn 2008) to evaluate the classification of T1 and T2 samples (see Supplementary material). The intra-host single nucleotide variants (iSNVs) mapped in Spike protein were investigated to potentially overlap with T cell S-reactive epitopes using *in silico* data as described in Supplementary material.

### 2.3 Consensus dataset collation and phylogenetic inference

We searched the Global Initiative on Sharing Avian Influenza Data (GISAID) database (https://www.gisaid.org) in mid-August for all complete genomes of SARS-CoV-2 collected between March and June in three Brazilian states: Rio de Janeiro (RJ), São Paulo (SP), and Minas Gerais (MG). We gathered 135 genome sequences to compose our phylogenetic dataset (GISAID accession numbers are available in Supplementary Table S7) and used the multiple alignment using fast Fourier transform algorithm to build the multiple sequence alignment from the resulting dataset (Katoh and Standley 2013). We estimated maximum likelihood phylogeny with this alignment of 201 genomes using a general time-reversible nucleotide substitution model (Tavaré 1986) with a proportion of invariable sites (GTR + I), selected by Modelfinder (Kalyaanamoorthy et al., 2017) in IQTree v.1.6.12 (Nguyen et al., 2015). Ancestral sequence reconstruction was implemented using the empirical Bayesian method in IQTree v.1.6.12. Branch support values were assessed by 1,000 replicates of ultrafast bootstrap approximation (Hoang et al., 2018). We assessed virus lineages for the whole dataset using Pangolin (https://pangolin.cog-uk.io) V 2.0.7 software (Rambaut et al., 2020) and checked our sequences for recombination using the full exploratory recombination method in RDP4 (Martin et al., 2015) and by the Phi-test approach (Bruen, Philippe, and Bryant 2006) in SplitsTree (Huson and Bryant 2006). We used Gblocks to select the most conserved regions of our multiple sequence alignment with default parameters (Castresana 2000). A unique block was selected with 29,476 sites, representing 98 per cent of the original alignment (flank positions: 215–29690).

## 3. Results

### 3.1 Study cohort

**Figure 1.**
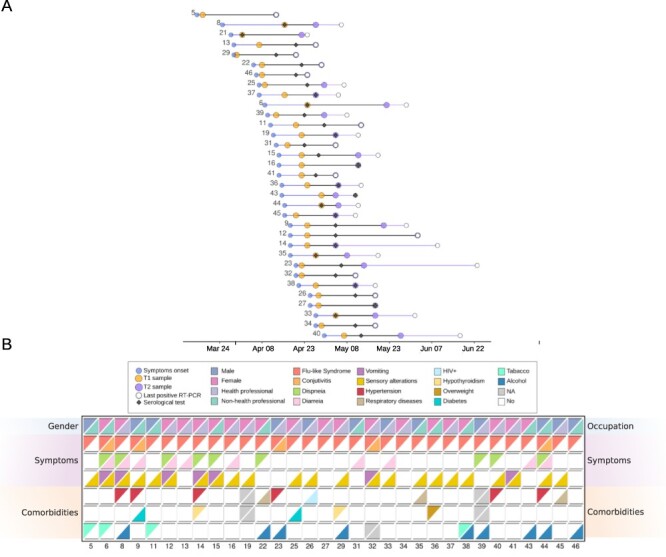
Characterization of patients with prolonged SARS-CoV-2 infection. (A) Time intervals between onset of patients’ symptoms (blue circles), first sample sequenced—T1 (orange circles), first positive serological test (black rhombuses), second sample sequenced—T2 (white circles), and last positive RT-PCR test (orange circles), respectively. The overlapping between dates are characterized by overlapping between circles as observed, for example, in some cases for white and purple circles. Blue lines represent the interval between onset of patients’ symptoms and first sample sequenced. Black lines indicate the difference between the two samples sequenced whereas in purple we show the difference between the second time sample and the last positive RT-PCR test. (B) Key clinical features for the patients analyzed in this study. Each row indicates two different features, first row: gender and occupation; second-to-fourth rows: symptoms, fifth-to-seventh rows: comorbidities. Patients are represented in the columns.

Twenty-one females and 12 males with mean ages of 39 ± 11 and 38 ± 9 years, respectively, were enrolled in this study. Most participants were health professionals (*n* = 19) or other workers (*n* = 7) from hospitals and clinics from the city of Rio de Janeiro (Supplementary Table S1; Fig. 1). A single patient was recruited from the State of Minas Gerais. We defined prolonged infection in our cohort when a patient remained positive for SARS-CoV-2 RNA in nasopharyngeal samples for at least 14 days after the symptoms onset. We sequenced samples collected at two time points for each patient (T1 and T2). T1 was the first positive RT-PCR sample after the onset of symptoms, and T2 the last sample with a Ct value < 35, which was required for sequencing. The mean interval between the two samples (T1 and T2) was 18 ± 7 days (range 5–39). The time interval since the onset of the symptoms and T1 ranged between 1 and 22 days (Supplementary Table S1; Fig. 1A).

Fifteen patients did not declare any preexisting health condition and, among those who declared comorbidities, hypertension was the most prevalent disorder (*N* = 6). All patients developed mild respiratory symptoms of COVID-19. Sensory changes, diarrhea, and vomiting were also observed in a few participants (Fig. 1B). A single patient, who reported hypertension and hypothyroidism, required hospitalization for two days. Serology tests showed that all participants were reagents for enzyme-linked immunosorbent assay anti-SARS-CoV-2 spike protein at T2; six of them were already positive at T1 (Fig. 1A). Overall, the different clinical and phenotype outcomes observed in our cohort may indicate a more diverse spectrum of biological mechanisms underlying prolonged infection.

### 3.2 Intra-host viral genetic diversity and transmission

**Figure 2.**
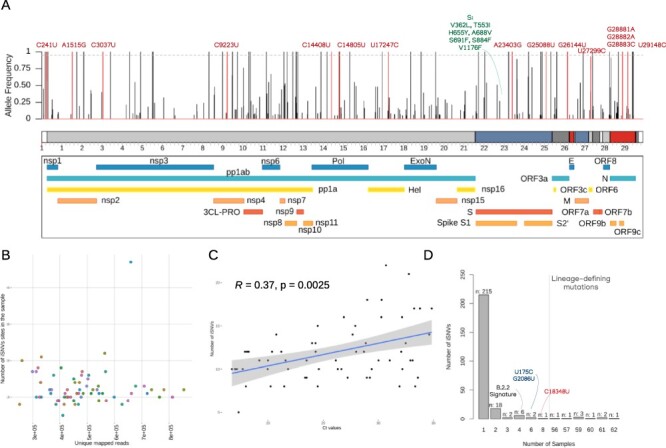
Intra-host genetic evaluation of SARS-CoV-2 genomes. (A) Distribution of iSNVs across the SARS-CoV-2 genome. Vertical line represents the within-host iSNV frequency targeting the protein products of the virus. In red, we showed the lineage-defining sites of each lineage identified in our samples. Dashed line indicates 95 per cent of allele frequency (B) Comparison of unique mapped reads versus number of iSNVs with MAF > 5 per cent of frequency identified in each of the 66 samples. (C) Spearman’s correlation tests between the Ct values and number of iSNVs. (D) Bar plot showing the distribution of iSNVs across the 66 samples.

Genetic screening for mutations in the full set of genomes revealed 253 iSNVs with allele frequency >5 per cent and <95 per cent spread across 9 open reading frames (ORFs) of SARS-CoV-2 (Fig. 2A and Supplementary Fig. S1; Supplementary Table S2). iSNVs with allele frequency below or above our threshold were listed as 0 per cent or 100 per cent, respectively. To rule out NGS data sequencing and processing steps as a spurious source of variation between T1 and T2, we only analyzed regions with a minimum coverage of 100 reads regardless of the presence of an alternative allele in both time points. Indeed, the mean number of unique mapped reads in each site was greater than 5,000 (Fig. 2B, Supplementary Fig. S1). We only observed one iSNV per genomic position, with nonsynonymous changes representing 174 (71 per cent) out of 244 variations in coding regions. Orf1ab harbored the majority of the iSNVs detected (*n* = 164) followed by the S protein (31), N (24), orf3a (7), M (5), orf7a (4), orf8 (4), orf6 (3), E (2), and noncoding regions (9). Nevertheless, when normalized by gene length, orf3 (b, c, and d), orf9, N protein, and orfs 6 and 7 showed the highest proportion of iSNVs (Supplementary Table S3). As expected, the Ct values were higher in T2 when compared to T1 (Supplementary Table S1; Supplementary Fig. S2). This behavior is widely consistent with the infection course of the disease once viral load (inversely proportional to Ct values) tends to decrease over time. Ct values were positively correlated to the number of iSNVs (Fig. 2C), highlighting that more variability was found at lower viral load (higher Ct values). We also found a nonlinear relationship between the number of iSNVs and the sampling interval since symptoms onset (Supplementary Fig. S2).

By comparing the mean number of iSNVs between the T1 (mean = 10.5) and T2 (mean = 14.7) sequences, we observed a significant difference (Wilcoxon test, *P* = 0.0002; SupplementaryFig. S2), mainly associated with the increase of nonsynonymous substitutions in T2 (Wilcoxon test, *P* = 0.0004). Such difference was found to be unrelated to genomic coverage (Spearman’s correlation ρ = −0.02, *P* = 0.8465). Thus, the variability shown is not caused by low mapping quality or miscalling variant issues. On average, nine sites were shared between both sequences in each subject, typically the lineage-defining mutations. T1 samples lost a mean of one exclusive iSNV that was not present in T2, whereas approximately five within-host mutations were acquired over time (Supplementary Table S4). The mean Minor Allele Frequency (MAF) per sample in T2 was significantly reduced with regard to T1, which suggests a gain of diversity driven by the acquisition of low-frequency iSNVs.

We then sought to estimate the magnitude of difference between T1 and T2 sequences by comparing the iSNV ratio per kb and per protein product along the SARS-CoV-2 genome. Both approaches revealed an elevated accumulation of variants (LogFC > 2) over time at eight main genomic windows, predominantly enriched at the 3ʹ-untranslated region (UTR) (Supplementary Table S3). These regions are responsible for encoding helicase, nsp15, nsp16, nsp10, Spike subunit S2, M, and orf7a proteins. Interestingly, alterations in Spike intersected known S-reactive CD4+ T cell peptides as well as T CD8+ predicted epitopes (See Supplementary material, Fig. S3). Accordingly, sites in helicase protein mainly affected the ATP-binding domain that donates the energy necessary to solve RNA secondary structures required during virus replication (Supplementary Fig. S3).

From the 45 iSNVs present in T1 and lost in T2, the highest abundance of variations occurred in the Orf1ab (*n* = 29), N (*n* = 6), and S (*n* = 4) proteins, respectively. Missense effects accounted for 30/45 iSNVs in this group. In Orf1ab, almost half of the sites targeted nsp3 (*n* = 5), nsp6 (*n* = 4), ExoN (*n* = 4), 3CL-PRO (*n* = 3), and Pol (*n* = 3). We hypothesized that the selection of these sites may be crucial to the continuous virus replication activity during prolonged infection. On the other hand, we detected 184 mutations acquired by T2 sequences that were absent in T1 (even considering that the positions had at least 100 reads of coverage). Two variations (S:S884F and C27389U) were shared between T2 samples of different individuals, probability due to linked transmission or convergent evolution events. Proportionally, most of the iSNVs target S protein (*n* = 22), helicase (*n* = 18), nsp3 (*n* = 15), and N (*n* = 15).

We noticed that most iSNVs (*n* = 215/253) were exclusively detected within-sample (Fig. 2D). From the 18 mutations only found in two samples, 16 were exclusively shared between T1 and T2 from the same subject (i.e. within-host). Five sites in this subgroup of samples showed dynamic scenarios of increase and decrease in within-host frequency over the course of the infection. Interestingly, only the C24213U (S884F) in S protein and the C27389U were found across T2 samples from two different individuals. Whereas C24213U had a within-host allele frequency of 14 per cent in T2 from Patient 45 and 100 per cent in T2 from Patient 28, C27389U showed a frequency of 8 per cent in T2 from Patient 27, and 100 per cent in Patient 6. Thus, both were low-frequency iSNVs in one subject and high-frequency iSNVs in the other, whether due to convergent events or linked transmission. Moreover, two iSNVs (synC11173U and synC5512U) were identified with a high frequency in the T1 and T2 from Patient 16 and were low frequency in Patients 13 and 37, respectively (Fig. 2D). Five out of the six iSNVs shared among four samples were lineage-defining mutations of B.2.2 (A1515G, C9223U, C14805U, U17247C, and G26144U), which matched the number of samples assigned to this lineage (Fig. 2D). The synC28253U is an exception, as it was found with high frequency in both sequences from Subject 40, with low frequency in T1 from Patient 37, and fixed in T2 from Patient 5. We also detected two iSNVs (U175C and G2086U) consistently shared with high frequency in T1 and T2 samples of Patients 14, 34, and 40 (Supplementary Table S2). A single variation synC18348U was shared among eight samples from T1 and T2 of the Subjects 9, 26, 29, and 38. Finally, the nine sites found in more than 50 samples were the lineage-defining mutations from B-derived viruses. Therefore, they were considered by us as fixed mutations instead of iSNVs. It is worth mentioning that B.1.1.28 and B.1.1.33 derived from B.1; thus, they shared common substitutions such as C241U, C3037U, C14408U, and A23403G. A total of 10 substitutions were assigned as lineage-defining mutations of B.1, B.1.1.28, and B.1.1.33 (Supplementary Table S5). No substantial difference was found between the expected and observed frequencies for these SNPs within the samples.

### 3.3 Phylogenetic analysis of consensus genomes

**Figure 3.**
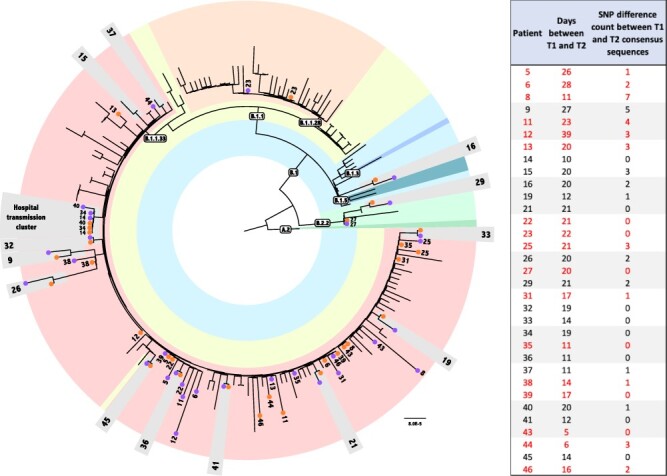
Phylogenetic analysis of prolonged samples. Maximum Likelihood tree obtained with Consensus dataset analysis under GTR + I model, containing a consensus genome for each time point of the 33 patients plus 135 populational samples. Populational sample names were excluded from the figure for clarity. Text boxes indicate the Pangolin lineage classification. A different color represents each sampled lineage in the tree. Numbers indicate each patient’s samples. Orange and purple circles represent T1 and T2 samples, respectively. Patients whose two time point samples are clustered are highlighted in light-gray boxes. Red numbers indicate patients that are not monophyletic in the haplotypes tree. Inset on the right side of the figure indicates the number of SNPs between the consensus sequences of T1 and T2 for each patient.

Reference-based viral genome assembly achieved, on average, 97 per cent genome coverage with a read depth greater than 2,000× in most of the regions sequenced. Similar results were found using a *de novo* assembly approach. To phylogenetically contextualize the 66 genomes generated in this study, we put together a dataset including 135 SARS-CoV-2 Brazilian genomes from Rio de Janeiro, São Paulo, and Minas Gerais states obtained from samples collected between March and June of 2020 (Supplementary Table S7). The maximum likelihood tree estimated for these 201 genomes is shown in Fig. 3.

The genomic sequences generated in our study were classified by Phylogenetic Assignment of Named Global Outbreak Lineages (PANGO) into four distinct lineages. Phylogenetic analysis generated concordant results. Most of them (58 out of 66) were classified as B.1.1.33 lineage. However, we also found viruses belonging to B.1.1.28 (*n* = 2), B.2.2 (*n* = 4), and B.1 (*n* = 2). T1 and T2 genomes for all patients were always concordant for the classification and assigned to the same lineage. To evaluate the possible occurrence of contamination in our sequencing, we investigated the presence of iSNVs at the lineage-defining mutations of each circulating lineage observed. On average, the lineage-defining sites of B.1.1.33, B.1.1.28, B.1, and B.2.2 had an allele frequency >95 per cent; thus, they were listed as 100 per cent (exception made for U27299C). Thus, no other alternative alleles were found at each position, unless those that characterize the lineages. For example, no iSNVs from B.1.1.33 lineage were detected in non-B.1.1.33 samples. The same results were observed for other lineage-defining sites in B.1.1.33 samples (Supplementary Figs S4; S5). Therefore, no evidence for contamination or coinfection by distinct lineages were observed in both time points. The monophyletic status of the sample pairs (T1 and T2) was recovered for 13 out of the 33 patients analyzed in this study, namely, Patients 9, 15, 16, 19, 21, 26, 29, 32, 33, 36, 37, 41, and 45 (Fig. 3). Ancestral sequence reconstruction indicates that the number of nucleotide substitutions in the branches supporting the monophyly of these patients vary between one and five.

One important aspect of the estimated tree is that it has 169 near-zero-length internal branches. All genome pairs that are not monophyletic are separated only by those not-supported branches. Therefore, considering that all patients enrolled in the study remained in social isolation until they had a negative PCR result and that their consensus sequences are more equally related to each other than to any other virus in the population, we have no evidence to consider the reinfection of those patients with other lineage.

We also found evidence of a transmission cluster for Patients 14, 34, and 40, who are health professionals at the same hospital (Fig. 3). All genomes (T1 and T2) from those patients share two iSNVs, U175C and G2086U (ORF1ab). Patient 14 presented symptoms first followed by 34 and 40, with a T1 sampling interval of 5 and 13 days, respectively. Indeed, T1 consensus sequences from these three patients, as well as T2 sequences from Patients 14 and 34, were identical. Patient 40’s T2 sample harbored only one substitution (U22119C) when compared to the other five consensus sequences previously described. Since the probability of having this shared variation as a result of recurrent mutations is too small, our results strongly suggest that they are the result of direct transmission between patients, indicating different events of infection with the same circulating virus in the hospital environment.

### 3.4 Dynamic alterations of mutational signatures in SARS-CoV-2 prolonged infection

**Figure 4.**
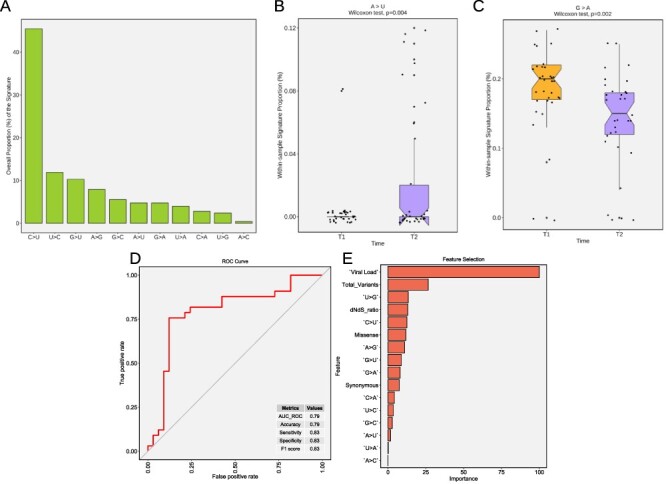
Differential mutational signatures and prolonged infection sample classification using machine learning models. (A) Distribution of the overall proportion of transitions and transversion in SARS-CoV-2 genomes in our study (B) A → U and (C) G → A proportions in samples from T1 and T2. (D) ROC curve showing a graphical representation of the relationship between sensitivity and specificity of the time point (T1 and T2) classification. The metrics table displays model performance. (E) Overall feature the importance and exhibits the most significant variables to separate the T1 and T2 classes.

Next, we examined the frequency of nucleotide changes at each iSNV site (MAF > 5 per cent) identified in the virus genome to investigate possible signatures associated with prolonged infection. Viral sequences exhibited a dynamic scenario of transitions and transversions mainly dominated by the C→U (45 per cent), U→C (12 per cent), and G→U (10 per cent) signatures (Fig. 4A). Although less common, longitudinal acquisition of iSNVs characterized by A→U transversion was significantly increased in T2 samples (Wilcoxon test, *P* = 0.004; Fig. 4B). On the other hand, the proportion of G→A significantly decreased over time (Wilcoxon test, *P* < 0.002; Fig. 4C; Supplementary Fig. S6). The other mutational signatures did not reach statistical significance between the groups. Multiple pairwise correlation tests showed that the decrease in G→A is strongly related to G→C mutations and negatively related to (G|C|A)→U (Supplementary Fig. S6). In addition, U↔C signatures also showed an inversely proportional relationship (Supplementary Fig. S6). Such discrepancy among the mutation signatures provides evidence of a complex interaction between signatures with decreasing Adenosine sites whereas Uracil increases. These findings reflect, as a fingerprint, the real-time evolutionary process occurring within-host.

Finally, given the differential accumulation of genomic marks over time observed in our analysis, we sought to distinguish long-term infection samples through a combination of features using a machine learning model. We then built a classification model that demonstrated that T1 and T2 genomes could be distinguished with 79 per cent accuracy. The receiver operating characteristic curve with area under the curve of 0.79, 83 per cent sensitivity, and 83 per cent specificity (Fig. 4D) showed a slight difference between the classified groups. This difference could be understood by analyzing the feature importance of the classification model (Fig. 4E). The most important features in T1 and T2 are related to viral load, total number of variants, dNdS ratio, U → G and C → U signatures, and missense mutations. The F1 score metric reached 83 per cent which indicates a balanced relation between precision and recall. The 83 per cent sensitivity suggests a slight difference in the classification of T1 and T2. Since no difference was observed comparing sensitivity with specificity, we considered the model’s performance acceptable for classification. The variance structure accurately shows the most important feature of the classification model.

## 4. Discussion

In this study, we analyzed 33 patients who remained RT-PCR positive for SARS-CoV-2 for over 2 weeks by performing a viral intra-host genomic analysis using a high-throughput sequencing approach. Our results demonstrate that SARS-CoV-2 may establish prolonged infection in the nasopharynx of seroconverted patients. These findings highlight the importance of PCR testing to ensure viral clearance and reduce the transmission of COVID-19. The phylogenetic analysis allowed the undeniable identification of 13 patients with long-term infection due to the monophyletic grouping between T1 and T2 consensus sequences. Even with growing evidence that SARS-CoV-2 reinfections may occur (To et al., 2020; Parry 2020), none of our patients had an inconsistent lineage assignment in T1 and T2. Thus, we have no clear indication that supports the hypothesis of reinfection or coinfection in our cohort. Moreover, genomic sequences from patients with prolonged infection were assigned to four different lineages suggesting that this is not a lineage-exclusive phenomenon and might be associated with multiple factors.

Among possible host-related factors, we found no evidence of clinical or phenotypic differences associated with the outcome once a wide range of symptoms and comorbidities were reported in the patients. On the other hand, generation of genomic diversity over the course of infection has been demonstrated as an important mechanism to establish virus persistence (Karim et al., 2021). By comparing two different time points from the same individual, we were able to track the acquisition and loss of variability during prolonged infection. Interestingly, Lythgoe et al. reported similar results comparing the T1 and T2 of 41 individuals without persistent infection with a mean of 6 days apart between the samples (Lythgoe et al., 2021). Here, the mean interval of time between the samples sequenced was 18 days apart, which may explain the higher intra-host diversity observed. Both studies detected more iSNVs at lower viral load; however, this could also be a result of stochastic sampling effects meaning that more variants may cross the minimum frequency threshold (Lythgoe et al., 2021). The increase in the iSNV number matched the T2 samples, which presented higher Ct values due to infection resolution at the upper respiratory tract. The cumulative virus diversity over the course of infection may also be caused by RNA polymerase errors.

Indeed, part of the most common mutational profiles identified such as G → U and U → C have been previously associated with RdRp mutational error spectrums (Smith et al., 2013). Nonetheless, other distinct mutational signatures may reflect the host RNA-editing enzyme activities on the viral genome as a cell defense mechanism (Wei et al., 2020). For instance, high levels of C → U and G → A described may be caused due to APOBEC-mediated deamination (Niavarani et al., 2015). We noticed a decrease in G → A and C → A mutations in T2 samples possibly because of nonsense-mediated decay pathway recognition of premature stop codon induced by APOBEC editing (Chester et al., 2003). Adenosine sites are also the target of A-to-I editing meditated by the Adenosine Deaminases Acting on RNA (ADAR) enzyme. Thus, we cannot exclude the possibility of fewer adenosine-mutated sites at the second time point mediated by ADAR activity, which seems to be more effective in restricting viral propagation than APOBEC (Di Giorgio et al., 2020). Both RNA-editing enzymes have already been described as antiviral factors stimulated by interferon in many other RNA viruses.

We observed a differential accumulation of variants in structural and non-structural proteins such as S and helicase that may play an important role in the prolonged infection. Helicase is a conserved protein responsible for the resolution of RNA secondary structures during the replication cycle of the virus (Jia et al., 2019). Targeting helicase activity using inhibitors is a potential candidate for COVID-19 therapy (Habtemariam et al., 2020). In addition, most iSNVs in Spike protein mapped in T cell reactive and predicted epitopes, according to the Immune Epitope Database (IEDB) database, some of the mutated antigen sequences identified in our analysis may have differences in binding affinity for epitopes to Major Histocompatibility Complex (MHC)-I and MHC-II. These variations could ultimately be associated with a mechanism of escaping the host’s immune response.

Recent studies demonstrated the acquisition of two advantageous SNPs for the virus (N501Y and E484K) in the receptor-binding domain (RBD) of Spike protein during persistent infection in an immunocompromised patient (Choi et al., 2020; Karim et al., 2021). Both mutations have been associated with high transmissibility and escape from neutralizing antibodies against SARS-CoV-2. N501Y and E484K were found as lineage-defining mutations in novel viral variants spread in the UK, South Africa, and Brazil (Tegally et al., 2021; Voloch et al., 2021; Silva Francisco et al., 2021; Volz et al., 2021; Faria et al., 2021). We observed acquisition of mutations over time in important residues of the RBD region previously associated with viral infectivity (Li et al., 2020). Therefore, genomic evolution during prolonged infection of SARS-CoV-2 might shed light on emergence and spread of novel SARS-CoV-2 variants. The prolonged duration of SARS-CoV-2 in some patients with detectable immunoglobulin G anti-Spike, like those investigated here, could select variants resistant to antibodies and contribute to novel variant emergence.

In conclusion, our study suggests routes for intra-host genomic evolution of SARS-CoV-2 during prolonged infection. We observed that most intra-host variations in SARS-CoV-2 present individual specificity and were not longitudinally transmitted, indicating that they probably were not adaptative. Only few iSNVs were fixed and shared among different subjects. The RNA-editing enzyme activities of the innate immune system of the human host could be associated with the temporal accumulation of iSNVs along the SARS-CoV-2 genome. Whether the upregulation in the mutation rate of Spike and helicase is an adaptive feature still needs further investigation. Our findings have potentially exploitable implications for public health decisions during the management of the COVID-19 pandemic as well as therapeutic uses that should be investigated.

## Data availability

NGS data generated in our study are publicly available in SRA-NCBI (https://www.ncbi.nlm.nih.gov/sra), Bioproject accession PRJNA675840. Genome sequences are also deposited in Gisaid (www.gisaid.org) and the access identifiers are listed in Supplementary Table S1.

## Supplementary Material

veab078_Supp
