# Supplementary material for: Intra-host evolution during SARS-CoV-2 prolonged infection
Source: Virus Evol. 2021 Sep 29;7(2):veab078. doi: 10.1093/ve/veab078 (PMC8500031; doi:10.1093/ve/veab078)
Supplement: veab078_Supp [file veab078_supp.zip › Figure_S5.pdf]

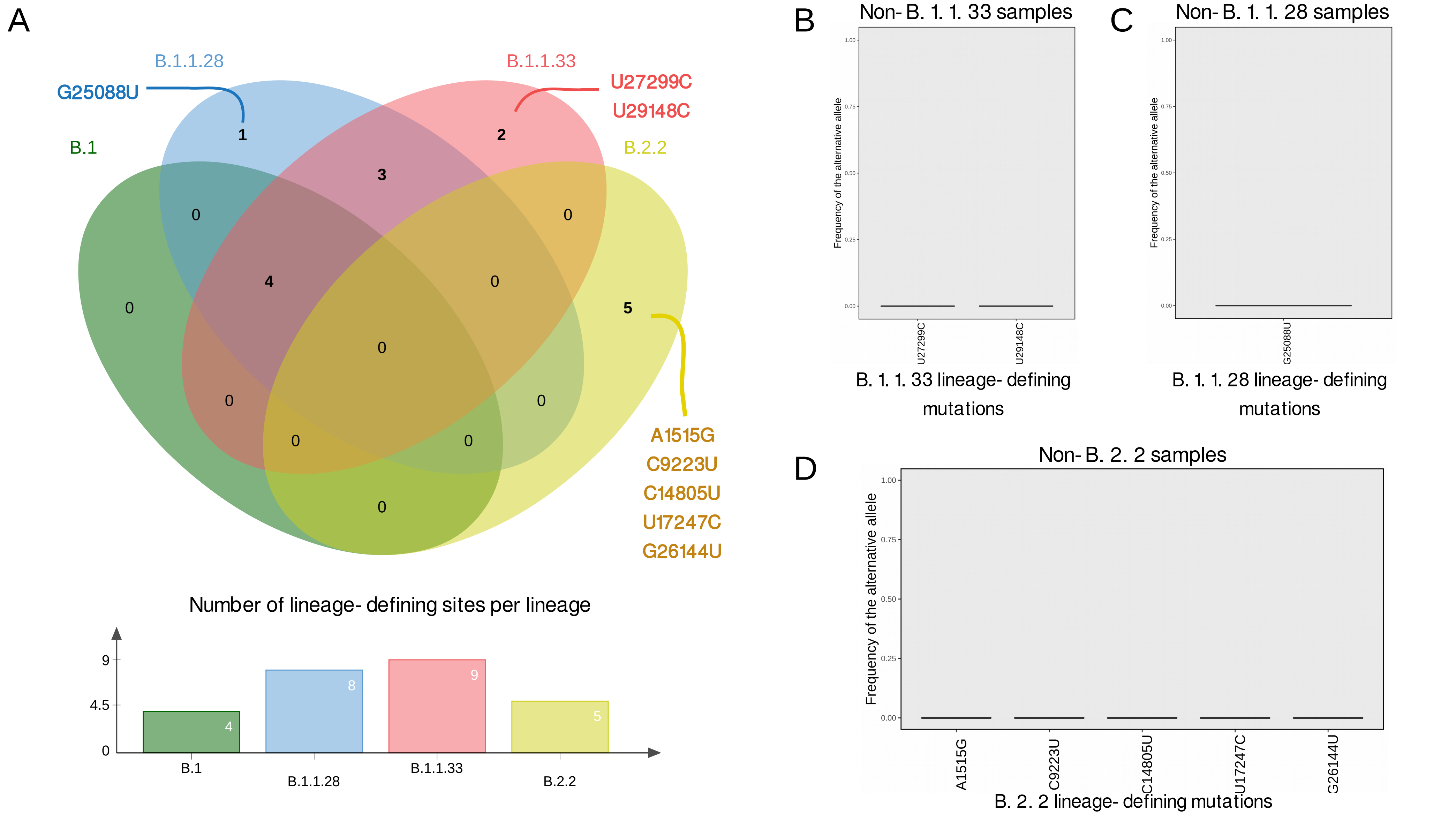
A
B.1.1.28
B.1.1.33
G25088U
U27299C
U29148C
B.1
B.2.2
1
2
3
0
0
4
0
0
5
0
0
0
0
0
0
A1515G
C9223U
C14805U
U17247C
G26144U
Number of lineage- defining sites per lineage
9
4.5
0
4
8
9
5
B.1
B.1.1.28
B.1.1.33
B.2.2
B
Non- B. 1. 1. 33 samples
Frequency of the alternative allele
1.00
0.75
0.50
0.25
0.00
U27299C
U29148C
B. 1. 1. 33 lineage- defining mutations
C
Non- B. 1. 1. 28 samples
Frequency of the alternative allele
1.00
0.75
0.50
0.25
0.00
G25088U
B. 1. 1. 28 lineage- defining mutations
D
Non- B. 2. 2 samples
Frequency of the alternative allele
1.00
0.75
0.50
0.25
0.00
A1515G
C9223U
C14805U
U17247C
G26144U
B. 2. 2 lineage- defining mutations
