# Supplementary material for: Intra-host evolution during SARS-CoV-2 prolonged infection
Source: Virus Evol. 2021 Sep 29;7(2):veab078. doi: 10.1093/ve/veab078 (PMC8500031; doi:10.1093/ve/veab078)
Supplement: veab078_Supp [file veab078_supp.zip › Supplementary_Material_Final_Version.docx]

**Supplementary Material**

**Intra-host evolution during SARS-CoV-2 prolonged infection**

Carolina M Voloch^1,*^, Ronaldo da Silva Francisco Jr^2,*^, Luiz G P de Almeida^2,*^, Otavio J. Brustolini^2,*^, Cynthia C Cardoso^1^, Alexandra L Gerber^2^, Ana Paula de C Guimarães^2^, Isabela de Carvalho Leitão^3^, Diana Mariani^1^, Victor Akira Ota^4^, Covid19-UFRJ Workgroup, LNCC-Workgroup, Cristiano X Lima^5,6^, Mauro M Teixeira^7^, Ana Carolina F Dias^6,7^, Rafael Mello Galliez^4^, Débora Souza Faffe^3^, Luís Cristóvão Pôrto^8^, Renato S Aguiar^9^, Terezinha M P P Castiñeira^4^, Orlando C. Ferreira^1^, Amilcar Tanuri^1^, Ana Tereza R de Vasconcelos^2,±^

***These authors contributed equally to this article.**

**^±^Correspondence**

# Workgroup Members

**Covid19-UFRJ Workgroup**

Alice Laschuk Herlinger, Aliny dos Santos Carvalho, André Felipe Andrade dos Santos, Anna Carla Pinto Castiñeiras, Átila Duque Rossi, Bianca Isabelle Barreto Teixeira, Bianca Ortiz da Silva, Bruno Clarkson, Bruno Eduardo Dematté, Camila de Almeida Velozo, Camila Nacif, Camille Victória Leal Correia de Silva Caroline Macedo Nascimento, Carolyne Lalucha Alves L. da Graça, Cassia Cristina Alves Gonçalves, Cíntia Policarpo, Ekaterini Simões Goudouri, Elaine Sobral da Costa, Elisangela Costa da Silva, Enrico Bruno Riscarolli, Érica Ramos dos Santos Nascimento, Fabio Hecht Castro Medeiros, Fábio Luís Lima Monteiro, Fernanda Leitão dos Santos, Fernando Luz de Castro, Filipe Romero Rebello Moreira, Francine Bittencourt Schiffler, Gabriela Bergiante Kraychete, Gabriele Silveira da Cunha, Gisely Novaes Borges da Cunha, Guilherme Sant'Anna de Lira, Gustavo Peixoto Duarte da Silva, Harrison James Westgarth, Helena D'Anunciação de Oliveira, Helena Keito Toma, Helena Toledo Scheid, Huang Ling Fang, Inês Corrêa Gonçalves, Ingrid Camelo da Silva, Isabela Labarba Carvalho de Almeida, Jessica Maciel de Almeida, Joissy Aprigio de Oliveira, Juliana Cazarin de Menezes, Juliana Tiemi Sato Fortuna, Karyne Ferreira Monteiro, Kissyla Harley Della Pascoa França, Laura Zalcberg Renault, Lendel Correia da Costa, Leticia Averbug Correa, Liane de Jesus Ribeiro, Lídia Theodoro Boullosa, Liliane Tavares de Faria Cavalcante, Luana dos Santos Costa, Lucas Matos Millioni, Luciana Jesus da Costa, Luiza Mendonça Higa, Marcela dos Santos Durães, Marcelo Amaral de Souza, Marcelo Calado de Paula Tôrres, Mariana Freire Campos, Mariana Quinto, Mariane Talon de Menezes, Marisa Souza Correia, Mateus Rodrigues de Queiroz, Matheus Augusto Calvano Cosentino, Mayla Gabryele Miranda de Melo, Mirela D'arc Ferreira da Costa, Pedro Henrique Costa da Paz, Raissa Mirella dos Santos Cunha da Costa, Raquel Fernandes Coelho, Richard Araujo Maia, Rodrigo de Moraes Brindeiro, Romina Carvalho Ferreira, Sérgio Machado Lisboa, Thamiris dos Santos Miranda, Victoria Cortes Bastos, Viviane Guimarães Gomes.

**LNCC-Workgroup**

Luciane Prioli Ciapina, Rangel Celso Souza, Éllen dos Santos Correa, Bruno Zonovelli da Silva, Amanda Araújo Serrão Andrade, Leandro Nascimento Lemos, Guilherme Cordenonsi da Fonseca.

**List of supplementary tables:**

**Table S1 -** Patient and sample information.

**Table S2 -** List of intra-host single nucleotide variants (iSNVs) with allele frequency >5% and <95% (iSNVs below or above these thresholds are listed as 0% or 100%, respectively).

**Table S3 -** Per gene and per protein mutation ratio comparison between T1 and T2.

**Table S4 -** Summarization of iSNVs across sequenced samples.

**Table S5** **-** Frequency of lineage-defining mutation across the samples.

**Table S6** **-** Spike mutations and epitopes analysis.

**Table S7 -** GISAID accession numbers for samples and acknowledgment table.

# Supplementary Methods

## Enzyme-linked immunosorbent assay

Microtitre plates (Immulon 2 HB) were coated with a trimeric spike proteins at 4 μg per ml in 100 mM sodium carbonate-bicarbonate buffer (pH 9.6) and incubated overnight at 4ºC. Excess protein was removed by washing five times with PBS + 0,05% Tween-20 (PBST, Sigma) and unbound sites blocked with 5% BSA in PBST. Samples were added at a 1/50 dilution in PBST + 2% BSA, followed by incubation at 37ºC/1 hour. Plates were washed five times with PBST then incubated for one hour at room temperature with polyclonal anti-human IgG antibody conjugated to HRP (Promega). Plates were again washed five times with PBST followed by addition of the chromogenic substrate TMB (Sigma) for 10 minutes and reaction stopped with 1N sulphuric acid. Absorbance was read at 450nm with an ELISA microplate reader (Biochrom Asys). A positive control specimen (from a known COVID-19 patient) in simplicate and a negative control (pre-epidemic plasma sample) in triplicate were added to every assay plate for validation and cut-off determination. Results were expressed as reaction of sample optical density value divided by the assay cutoff.

## Library Preparation and Sequencing

Total RNA from SARS-CoV-2 positive samples was converted to cDNA using the SuperScript IV First-Strand Synthesis System (Thermo Fisher Scientific, USA). Viral whole-genome amplification was performed according to the [Artic](https://artic.network/ncov-2019) Network protocol (https://artic.network/ncov-2019) using the SARS-CoV-2 primer scheme (V3). PCR products (pool A and B) were cleaned-up separately with AmpureXP beads (Beckman Coulter, UK), and then quantified using the Qubit dsDNA High Sensitivity assay (Life Technologies, USA). Equal amounts of each pool were combined and used to construct sequencing libraries with the TruSeq DNA Nano kit (Illumina, USA). The protocol was started at the end repair reaction, and followed as described by the manufacturer without any modifications. Library QC and quantification was performed using the High Sensitivity D1000 ScreenTape Assay on the 4200 TapeStation system (Agilent, USA). Libraries were sequenced in a MiSeq System with MiSeq Reagent Kit v3 (Illumina, USA) set to obtain 2x250 bp reads.

## Machine Learning Classification

Using the caret R package [(Kuhn 2008)](https://paperpile.com/c/UwnRX5/lI9c), we built a machine learning model based on the Random Forest algorithm optimized for tuning of parameters to evaluate classification of patient time points T1 and T2. To compose the feature matrix, we selected the following variables: viral load; missense and total variants; nucleotide substitution signatures; non-synonymous/synonymous ratio; non-coding and synonymous variants. The dataset was separated into two sets: 80% for training and 20% for testing. To evaluate model error, we applied 100,000 repeats of 10-fold cross-validation. Confusion matrix, ROC curve, and model performance metrics were calculated using the R package MLeval. Feature importance selection was applied using the caret function varImp for assigning scores and ranking features that indicate their relative importance when making a prediction. This approach has the advantage of using a model-based approach, which can incorporate the correlation structure between the predictors into the importance calculation [(Huynh-Thu et al. 2012)](https://paperpile.com/c/UwnRX5/ASTOG).

## Spike protein epitope analysis

To identify potential T cell S-reactive epitopes we performed an integrative analysis based on Immunology custom tracks available at UCSC Genome Browser. We downloaded and cross-referenced five different track data hubs using experimental and computational predicted T cell epitopes from T-React Epitopes [(Braun et al. 2020)](https://paperpile.com/c/UwnRX5/mEFfh), CD4+ and CD8+ IEDB Predictions [(Grifoni et al. 2020)](https://paperpile.com/c/UwnRX5/z4opy), Poran HLA I and HLA II [(Poran et al. 2020)](https://paperpile.com/c/UwnRX5/o4lGv/?locator_label=volume), and CD8 Rosetta MHC [(Poran et al. 2020; Nerli and Sgourakis 2020)](https://paperpile.com/c/UwnRX5/o4lGv+QDNhC). T-React Epitopes data was used as a reference resource due to the experimental validation of the peptides identified in the study. Computational predicted scores for each study were normalized using a min-max scaling feature. In each computational study, we only selected epitopes that mapped genetic variations identified in S protein. A mean score for the reference peptides was calculated in each prediction study. We then performed a binomial distribution test to compare the distribution of real peptides and the epitopes that carried mutations in our analysis.

## Characterization of SARS-CoV-2 spike protein T cell reactive and predicted epitopes

To explore the impact of spike variations on the host immune response or escape mutants, we intersected all low- and high-frequency iSNVs detected in Spike protein with known and predicted T cell epitopes. We found 11 iSNVs intersecting ten known S-reactive CD4+ T cell peptides in eight different patients (**Figure S3; Table S6**). Nine out of the 11 variants had a non-synonymous effect being mostly found in T2 samples, while only patient 37 harbored a variant in the T1 sample. Patients 16 and 25 had two and three variants mapping at least one reactive epitope, respectively. In addition, cross-referencing comparison with predicted CD4+ and CD8+ T cells revealed seven iSNVs overlapping six different T CD4+ epitopes (**Figure S3; Table S6**). Alterations in T CD8+ predicted epitopes were reported in 28 peptides in 62 samples (**Table S6**). The overwhelming majority of patients harbored the D614G substitution mapped in the YQDVNCTEV epitope. This peptide was also predicted as a putative HLA class I candidate binding peptide, together with 31 other regions with a high probability for immune recognition of SARS-CoV-2. Finally, 28 HLA class II candidate peptides had at least one variation across 27 patients (**Table S6**).

**List of supplementary figures:**

**Supplementary Figure 1. Filtration and characterization of iSNVs across the 66 samples sequenced. A)** Distribution of within-sample iSNVs frequencies and per site reads depth before and **B)** after filtering by a minimum of 100 reads of coverage and masked miscalled sites. **C)** Comparison between the reads depth and allele frequency in each iSNV site investigated. Each point represents a single genomic position in a single sample. Only iSNVs with MAF > 0.05 were included in the analysis. **D)** a Zoom in the distribution of within-sample iSNVs frequencies in sites with less than 2,000 unique mapped reads, and **E)** within-host frequency at the lineage-defining mutations SNPs.

**Supplementary Figure 2. Genetic diversity over the course of the infection. A)** Spearman’s correlation tests between Ct values and time since the symptoms onset and **B)** iSNVs number. **C)** Spearman’s correlation analysis of nucleotide diversity versus time since the symptoms onset and **D)** Ct values. **E)** Differential accumulation of iSNVs between T1 and T2. **F)** Correlation between within-sample G>A mutation proportions and infection time.

**Supplementary Figure 3. Accumulation of iSNVs in helicase and Spike proteins between T1 and T2. A)** Characterization of iSNVs targeting helicase protein. Zoom in helicase (nsp13) protein showing differential accumulation of genetic variants in T2 samples (orange circles) when compared with T1 (purple circles). Most iSNVs overlapped the ATP binding domain of the helicase protein. **B)** Characterization of iSNVs targeting spike protein. Zoom in spike protein showing (Top) iSNVs found in T1 samples in orange circles and variants in T2 in purple circles. (Middle) Density plot for each time and T cell (i) reactive epitopes, (ii) IEDB T CD4+ predicted epitopes, (ii) NetMHCpan4.0EL T CD8+ predicted epitopes, (iv) HLA-I candidate binding peptides, (v) HLA-II candidate binding peptides and (vi) T CD8+ epitopes predicted by Rosetta. Both regions harbored at least one iSNV in our patients. (Bottom) Normalized score for each predicted epitope represented by filled circles. Dashed lines represent the mean value of the score assumed by the S-reactive epitope in each computational approach considering the color matching.

**Supplementary Figure 4. Within-host frequencies of lineage-defining mutation. A-D**) shows the allele frequencies of B.1, B.1.1.28, B.1.1.33 and B.2.2 lineage-defining mutations in each individual. The circle represents the frequency of the alternative allele in each sample assigned to the lineage. Once the within-host frequency was ~98% for each allele, we observed an overlap between the circles. The variations were annotated according to the Wuhan-Hu-1 reference genome (NC_045512.2). No other alternative allele, unless those that characterize the lineages, were found at these sites. We then normalized sites with 95% by listing them as 100%. The colors in each panel represent the set of genomic variants in a given sample.

**Supplementary Figure 5. Investigation of contamination based on within-host allele frequency of lineage-defining sites.** Analysis of minor allele frequencies at distinguable lineage-defining sites from B.1.1.33, B.1.1.28 and B.2.2. A) Comparison between the substitutions classified as lineage-defining sites of each lineage identified in our samples. Exclusive substitutions in each lineage were highlighted and used as proxy to determine the possible presence of coinfection or contamination by distinct lineages within-sample. B) Frequency of exclusive B.1.1.33 substitutions in non-B.1.1.33 samples. C) Frequency of exclusive B.1.1.28 substitutions in non-B.1.1.28 samples. D) Frequency of exclusive B.2.2 substitutions in non-B.2.2 samples.

**Supplementary Figure 6. Mutational signature correlation analysis.** Pairwise Spearman’s correlation tests between the frequency of transitions and transversion in SARS-CoV-2 genome over time in each patient. Red squares indicate significant negative correlations (P < 0.05) whereas in blue we show positive correlations.

**References**

[Braun, Julian, Lucie Loyal, Marco Frentsch, Daniel Wendisch, Philipp Georg, Florian Kurth, Stefan Hippenstiel, et al. 2020. “SARS-CoV-2-Reactive T Cells in Healthy Donors and Patients with COVID-19.” *Nature*, July. https://doi.org/](http://paperpile.com/b/UwnRX5/mEFfh)[10.1038/s41586-020-2598-9](http://dx.doi.org/10.1038/s41586-020-2598-9)[.](http://paperpile.com/b/UwnRX5/mEFfh)

[Grifoni, Alba, John Sidney, Yun Zhang, Richard H. Scheuermann, Bjoern Peters, and Alessandro Sette. 2020. “Candidate Targets for Immune Responses to 2019-Novel Coronavirus (nCoV): Sequence Homology- and Bioinformatic-Based Predictions.” *SSRN*, February, 3541361.](http://paperpile.com/b/UwnRX5/z4opy)

[Hastie, Trevor, Saharon Rosset, Ji Zhu, and Hui Zou. 2009. “Multi-Class AdaBoost.” *Statistics and Its Interface*. https://doi.org/](http://paperpile.com/b/UwnRX5/aCFb)[10.4310/sii.2009.v2.n3.a8](http://dx.doi.org/10.4310/sii.2009.v2.n3.a8)[.](http://paperpile.com/b/UwnRX5/aCFb)

[Huynh-Thu, Vân Anh, Yvan Saeys, Louis Wehenkel, and Pierre Geurts. 2012. “Statistical Interpretation of Machine Learning-Based Feature Importance Scores for Biomarker Discovery.” *Bioinformatics*  28 (13): 1766–74.](http://paperpile.com/b/UwnRX5/ASTOG)

[Kuhn, Max. 2008. “Building Predictive Models in R Using the Caret Package.” *Journal of Statistical Software* 28 (1): 1–26.](http://paperpile.com/b/UwnRX5/lI9c)

[Nerli, Santrupti, and Nikolaos G. Sgourakis. 2020. “Structure-Based Modeling of SARS-CoV-2 peptide/HLA-A02 Antigens.” *bioRxiv : The Preprint Server for Biology*, March. https://doi.org/](http://paperpile.com/b/UwnRX5/QDNhC)[10.1101/2020.03.23.004176](http://dx.doi.org/10.1101/2020.03.23.004176)[.](http://paperpile.com/b/UwnRX5/QDNhC)

[Poran, Asaf, Dewi Harjanto, Matthew Malloy, Michael S. Rooney, Lakshmi Srinivasan, and Richard B. Gaynor. 2020. “Sequence-Based Prediction of Vaccine Targets for Inducing T Cell Responses to SARS-CoV-2 Utilizing the Bioinformatics Predictor RECON.” https://doi.org/](http://paperpile.com/b/UwnRX5/o4lGv)[10.1101/2020.04.06.027805](http://dx.doi.org/10.1101/2020.04.06.027805)[.](http://paperpile.com/b/UwnRX5/o4lGv)
