# Supplementary figures and images for: Intra-host evolution during SARS-CoV-2 prolonged infection
Source: Virus Evol. 2021 Sep 29;7(2):veab078. doi: 10.1093/ve/veab078 (PMC8500031; doi:10.1093/ve/veab078)

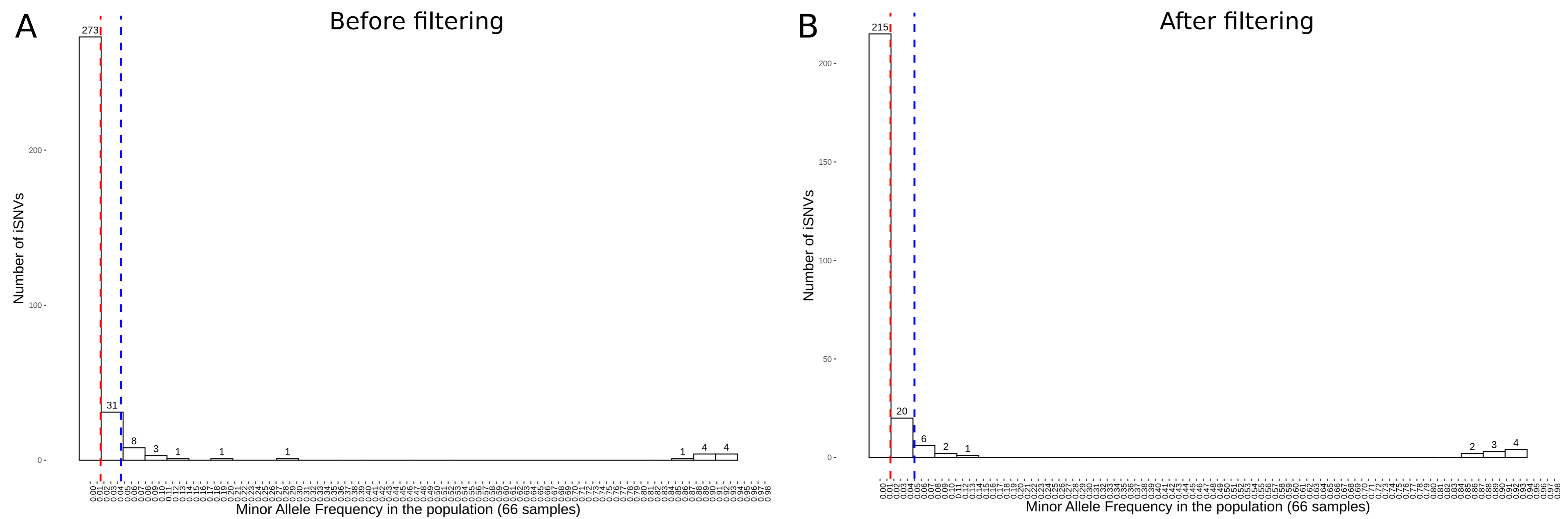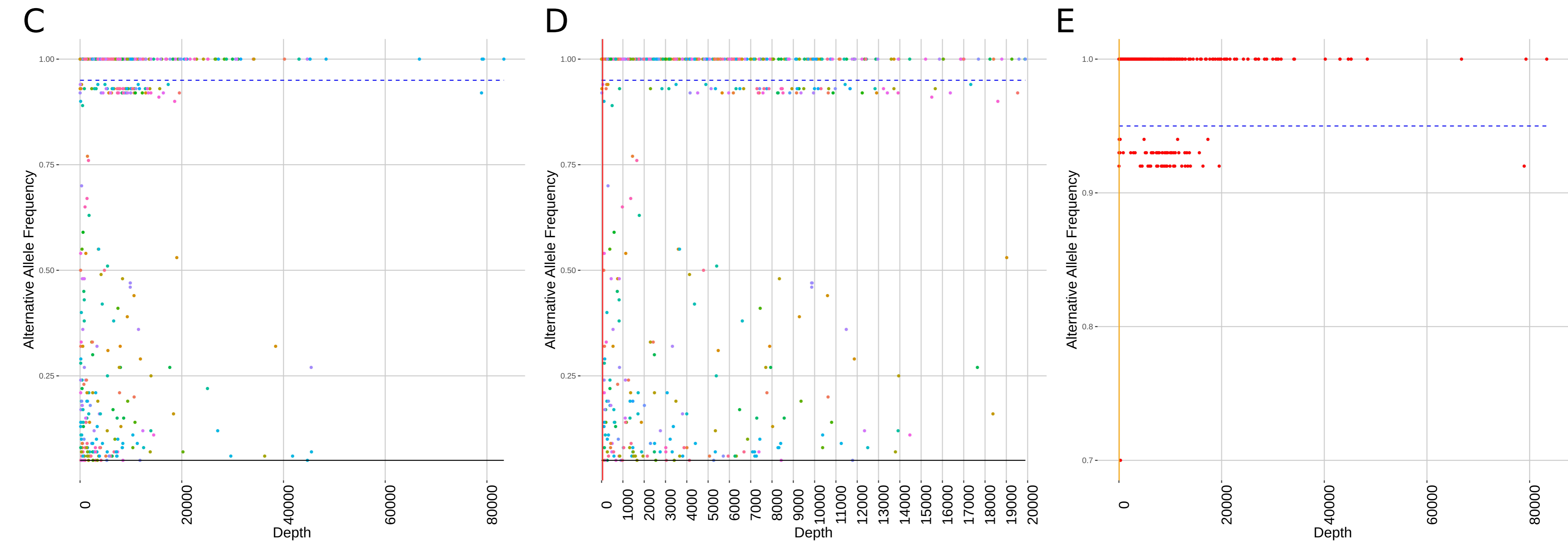

Supplement: veab078_Supp [file veab078_supp.zip › Figure_S1_R2.pdf]

A

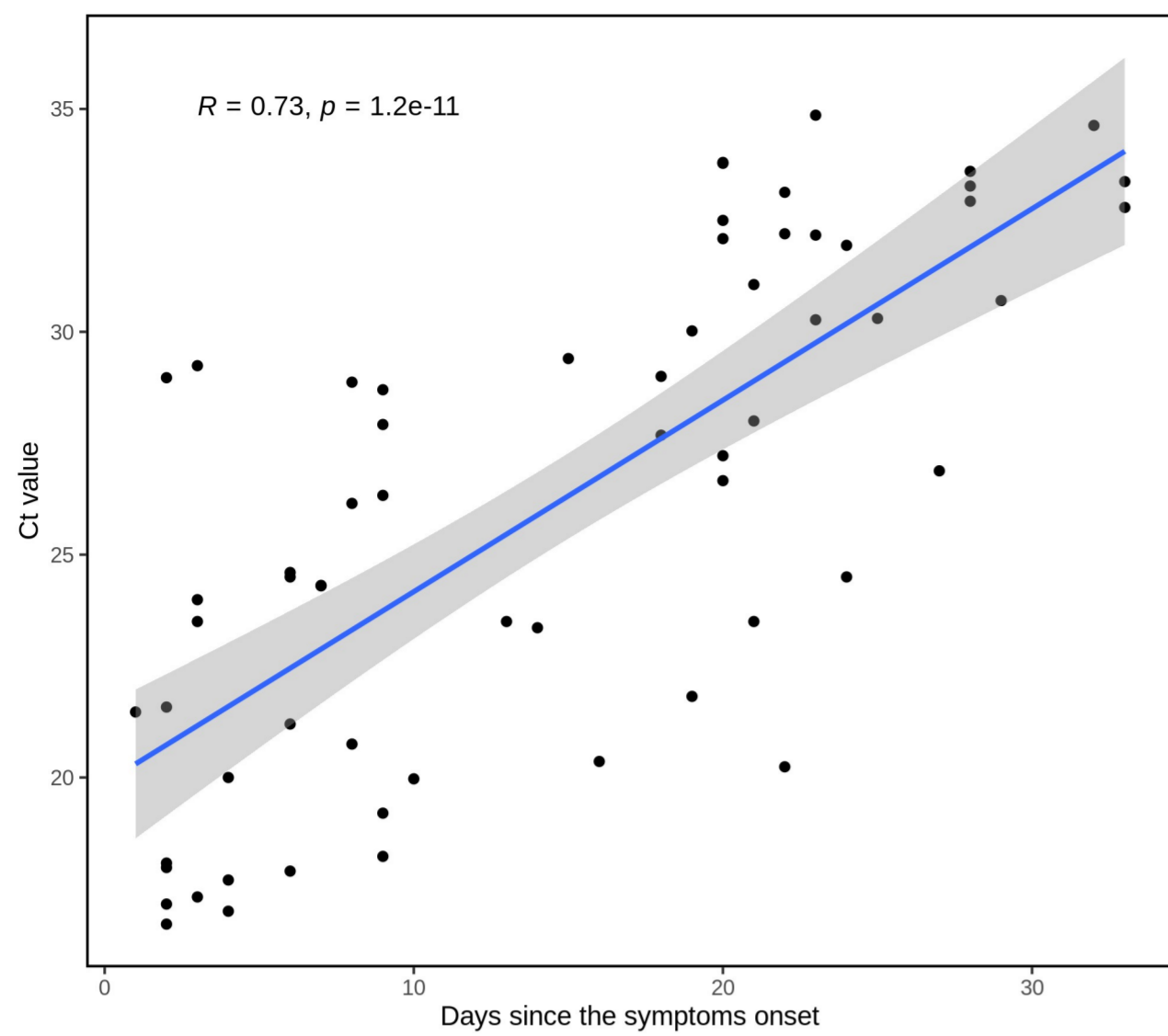

B

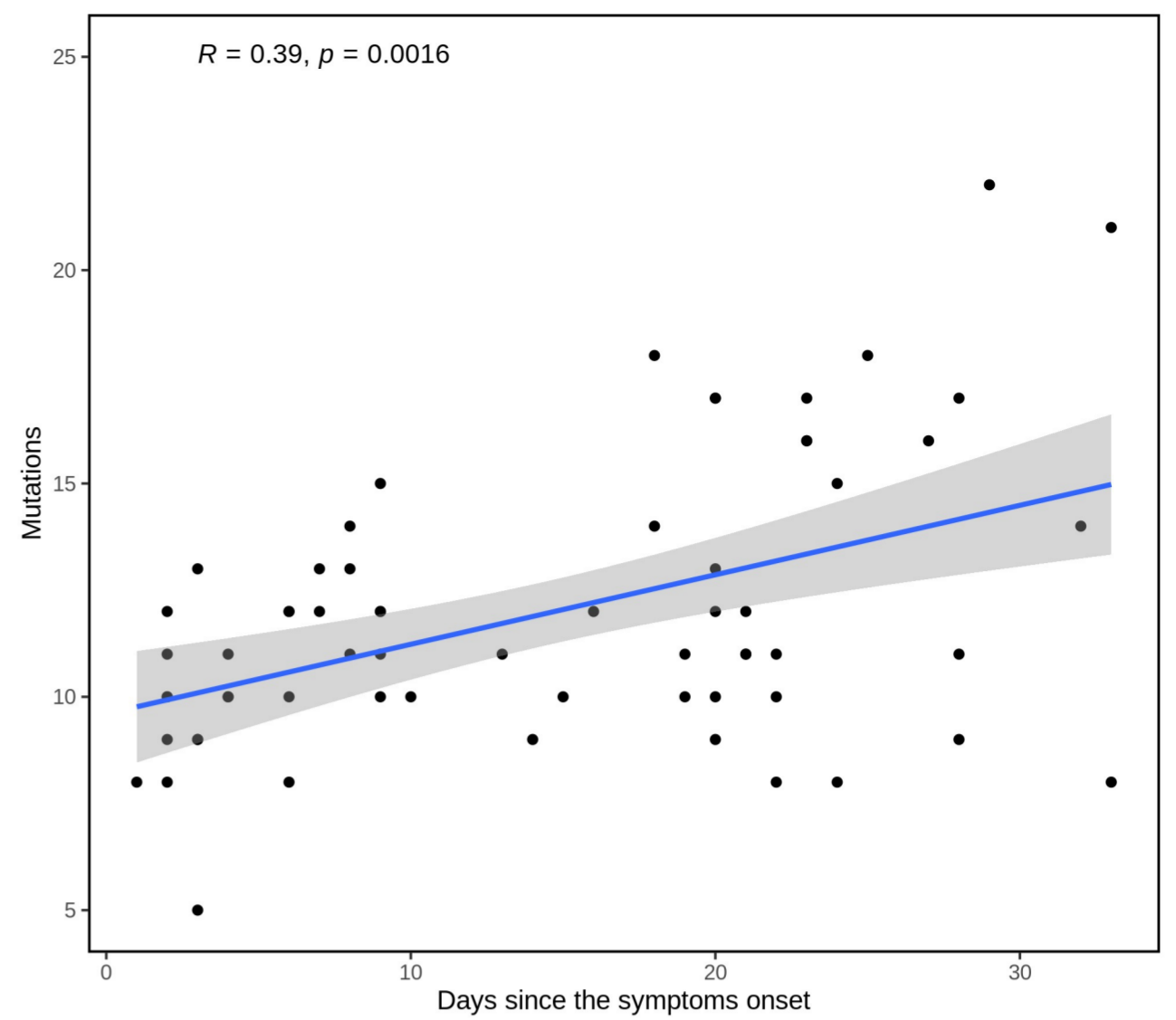

C

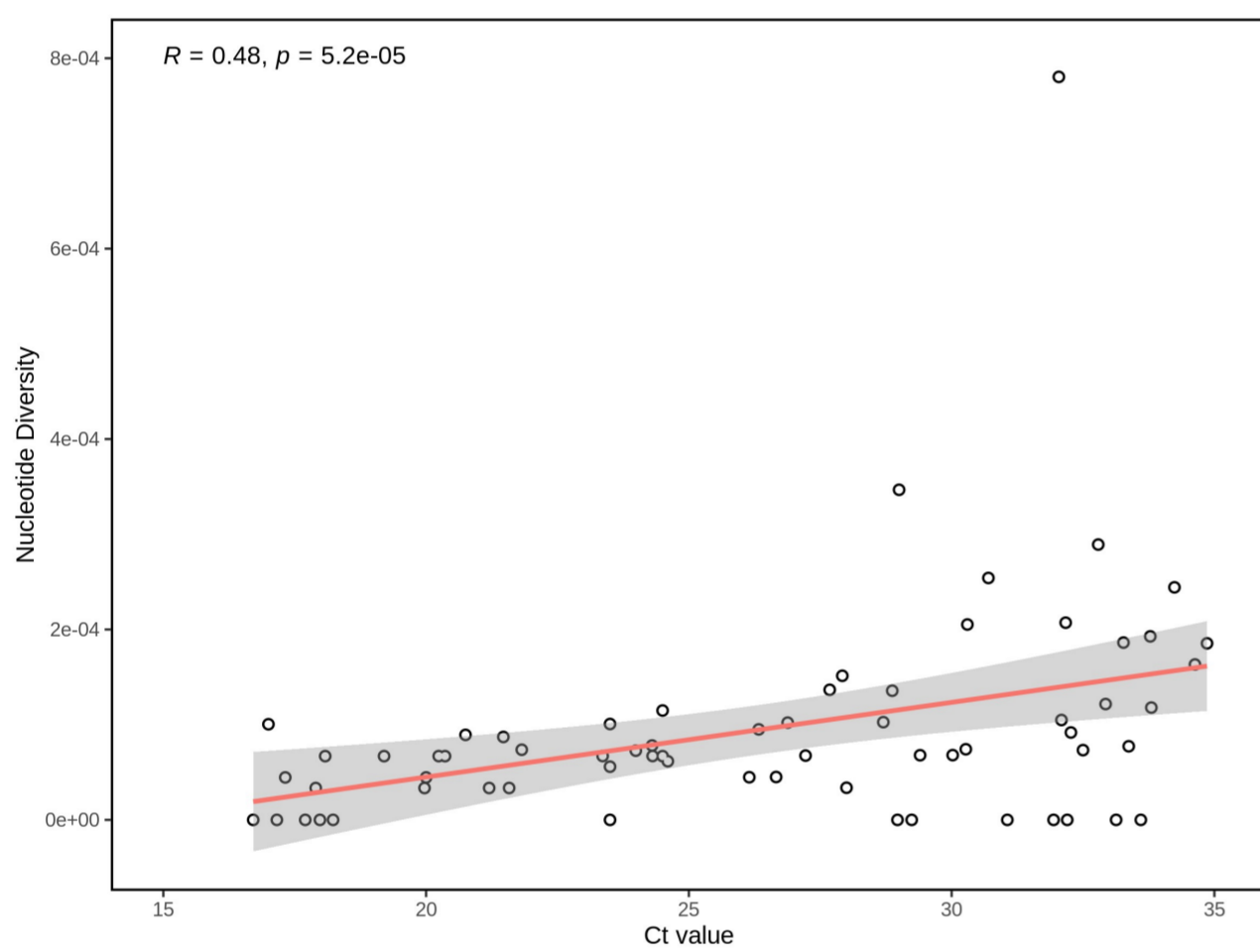

D

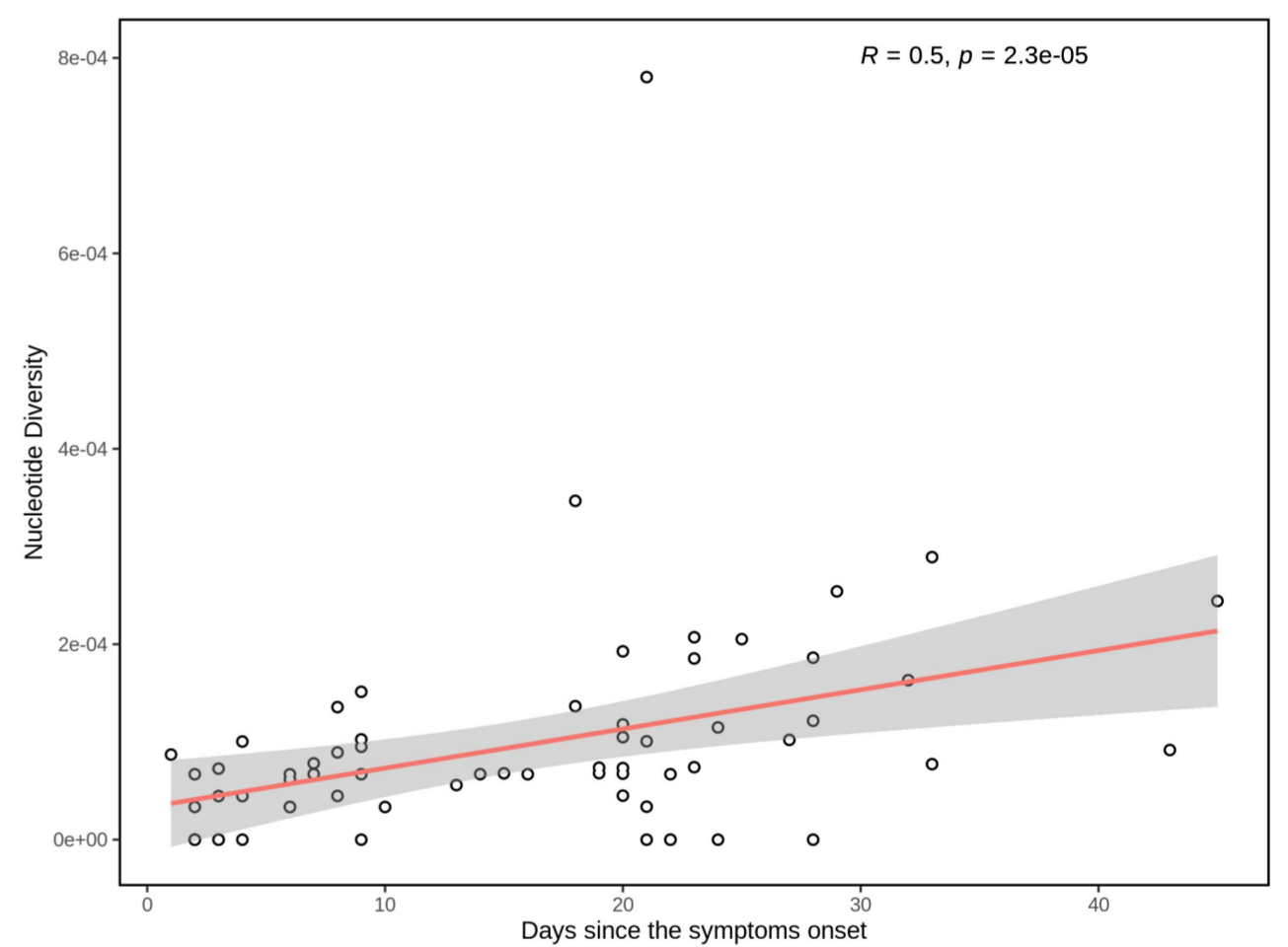

E

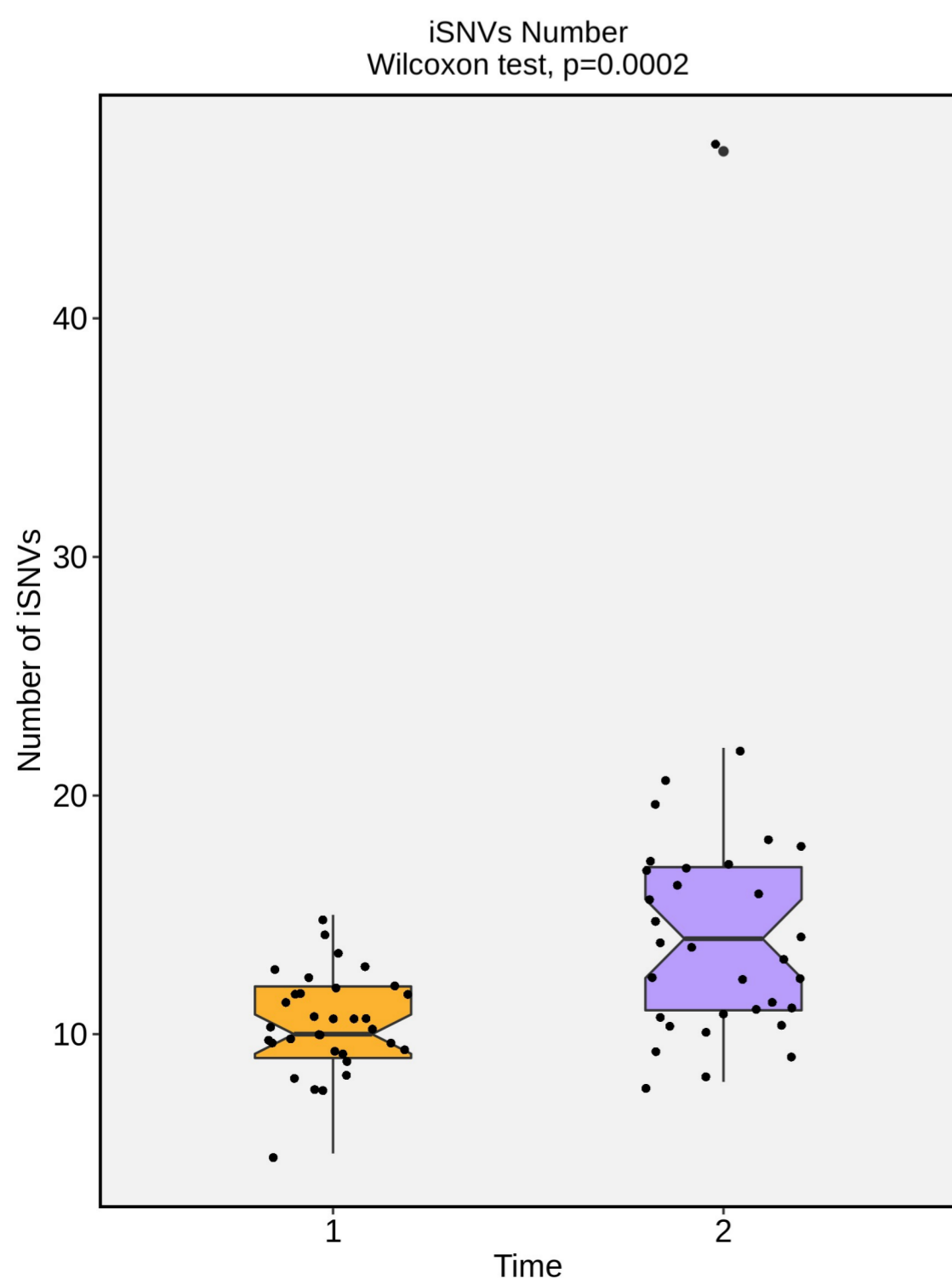

F

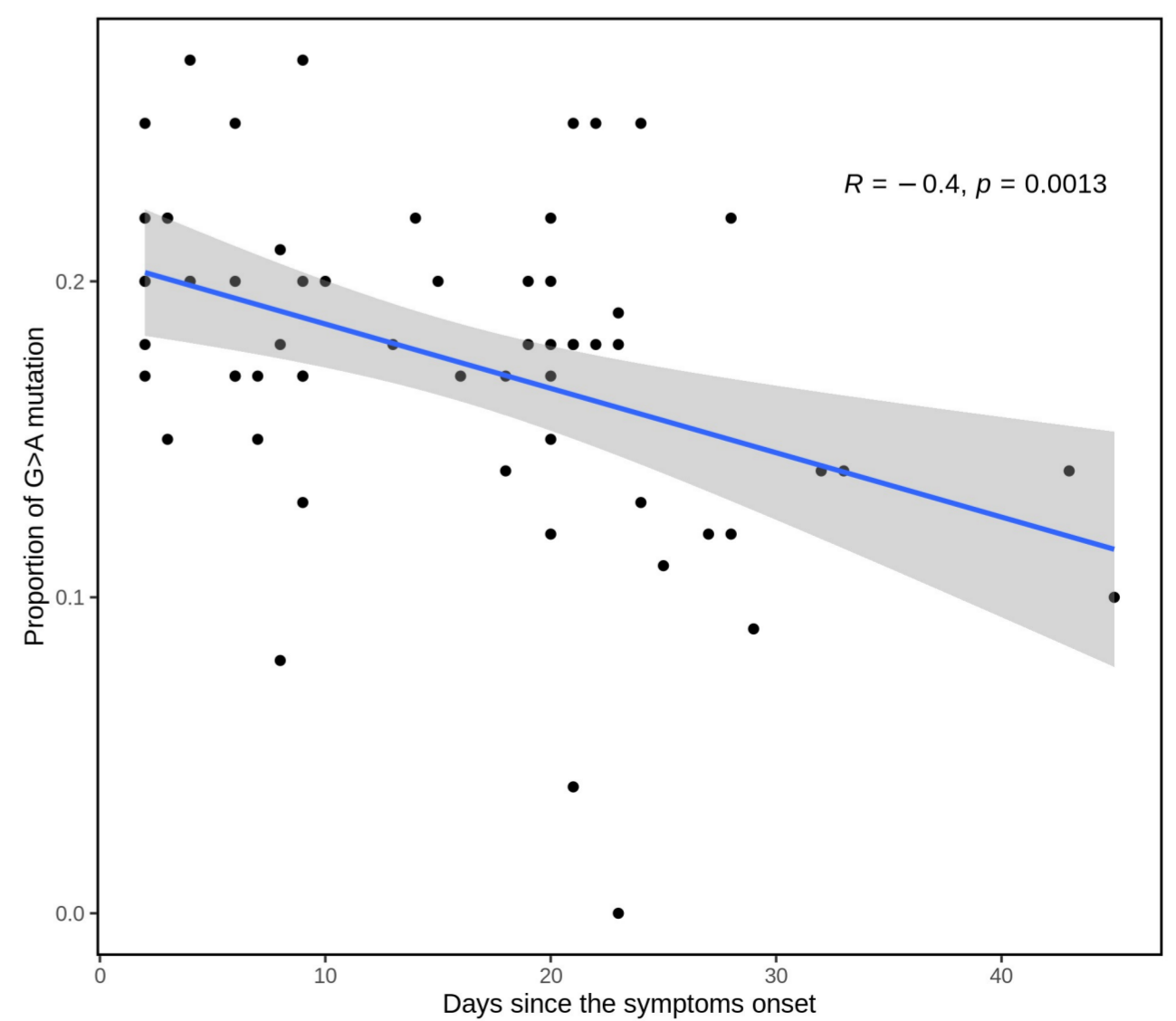

Supplement: veab078_Supp [file veab078_supp.zip › Figure_S2.pdf]

A

## Helicase Protein

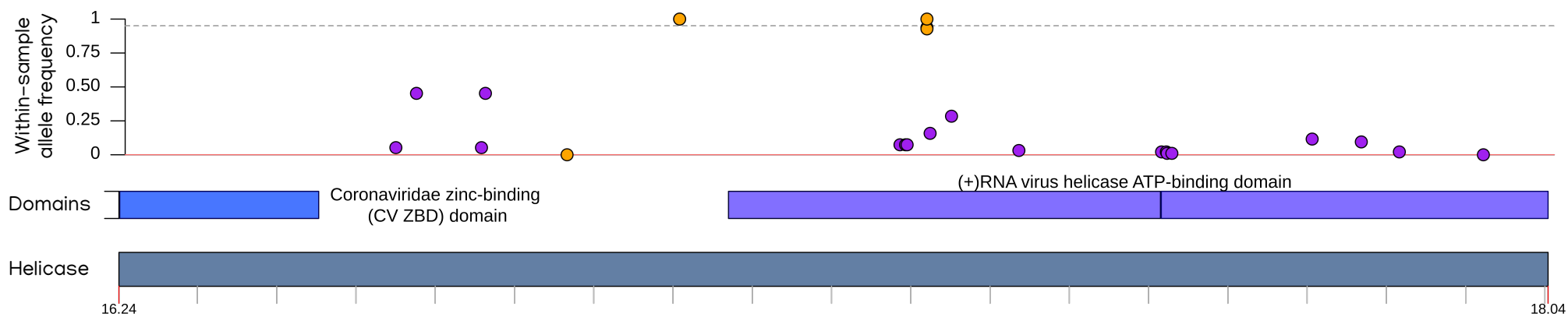

B

## Spike Protein

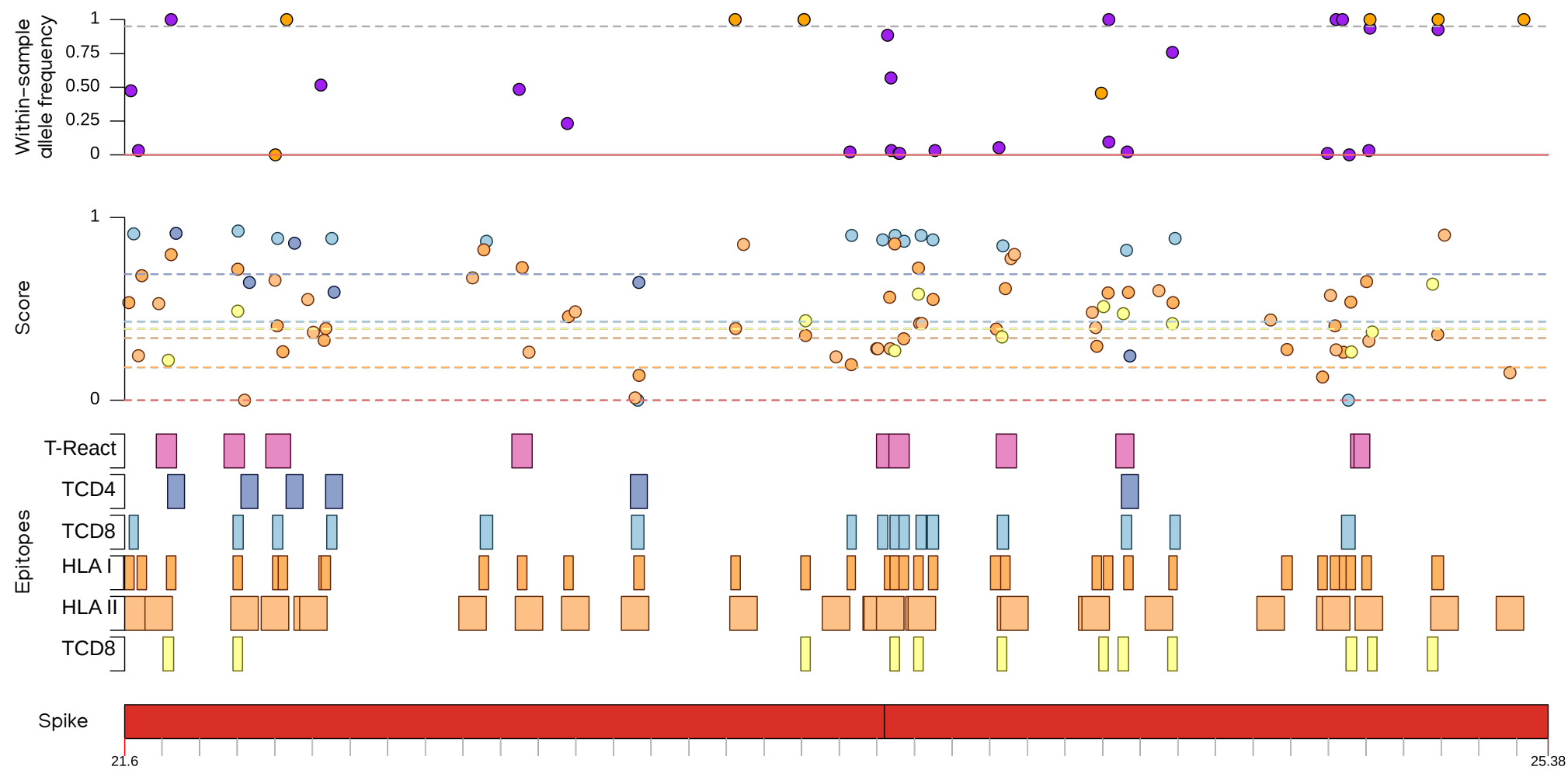

Supplement: veab078_Supp [file veab078_supp.zip › Figure_S3_R2.pdf]

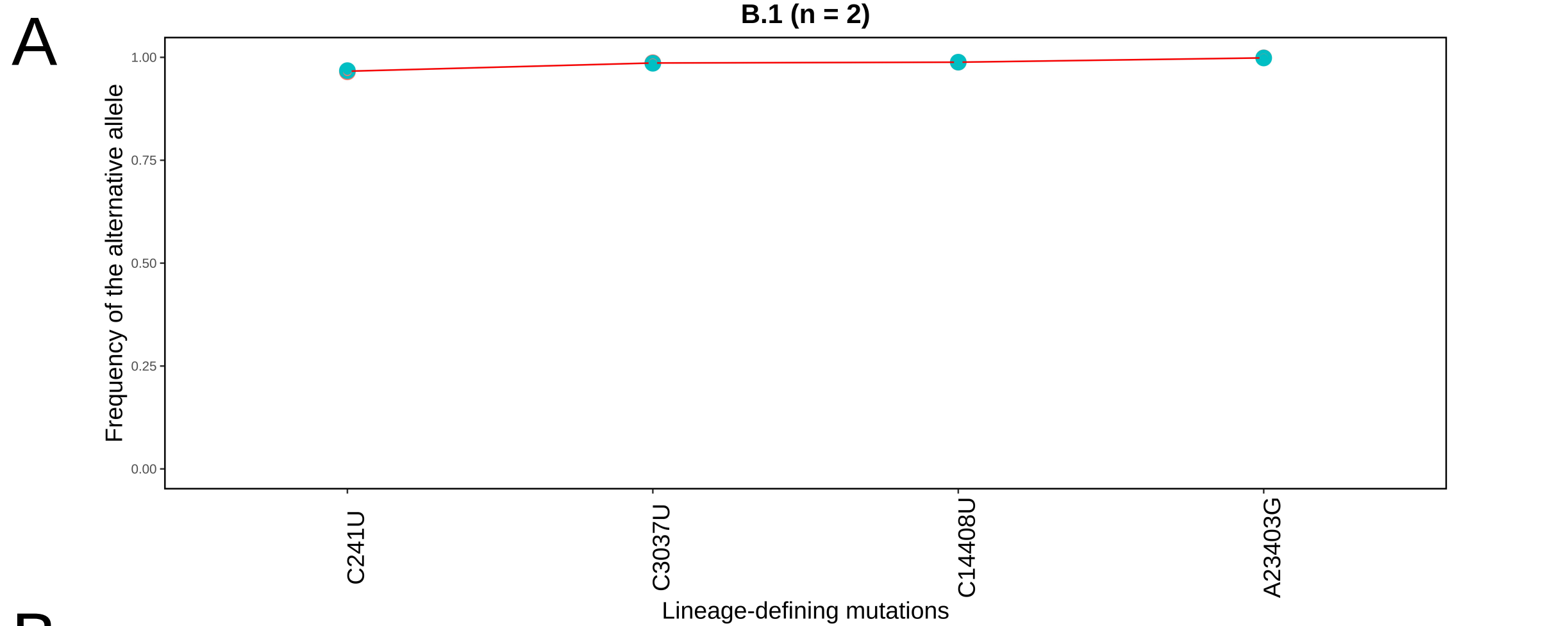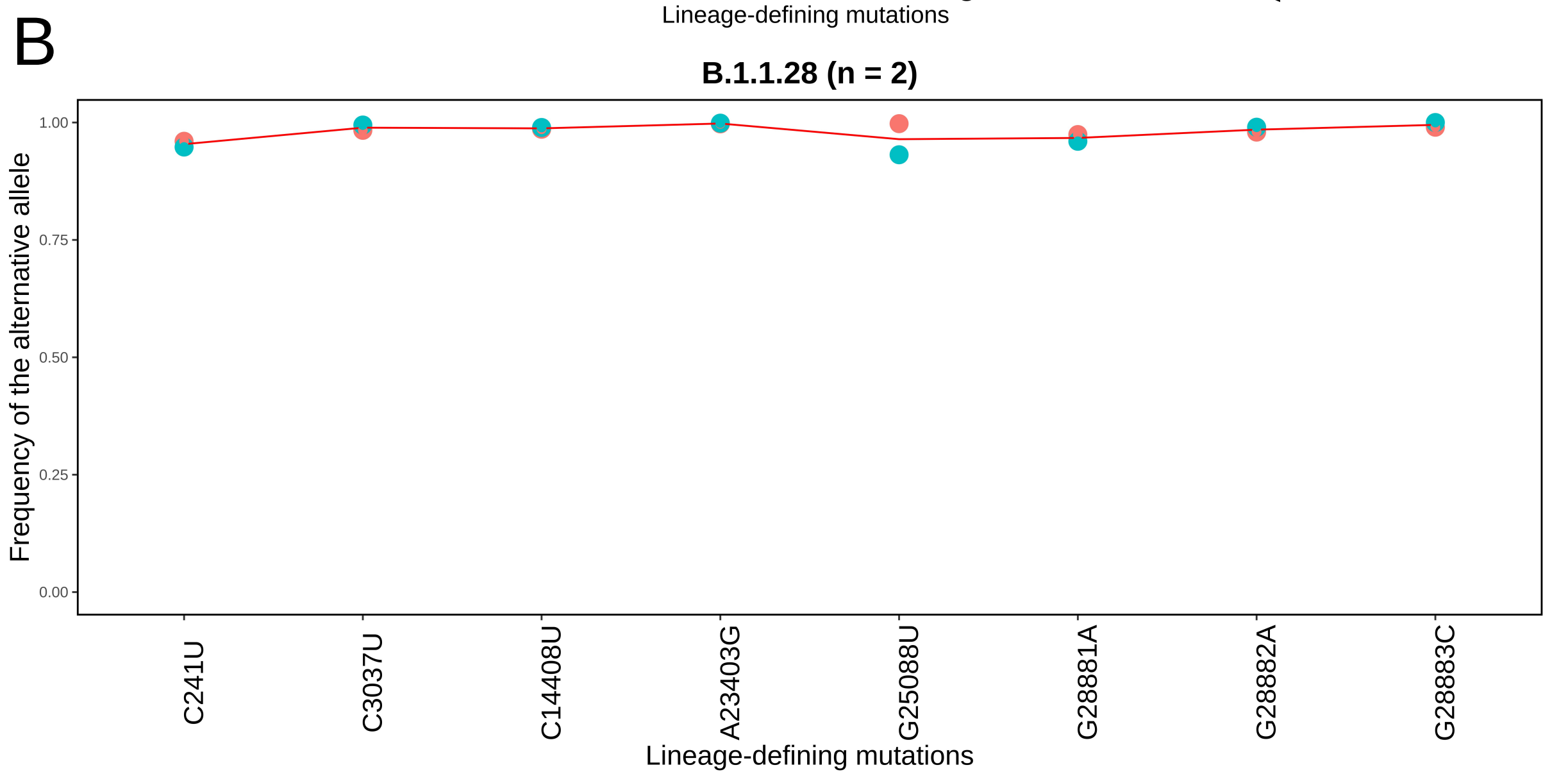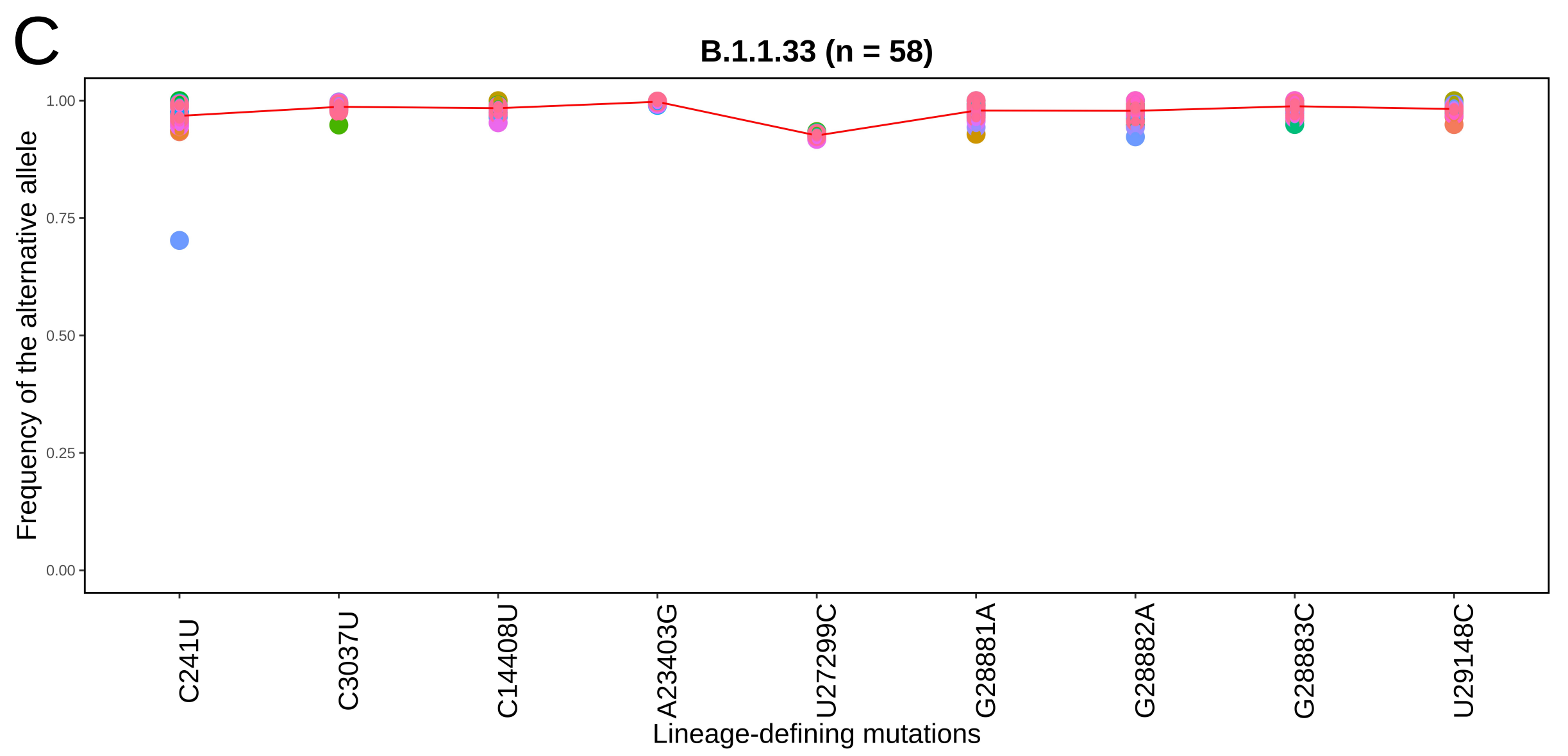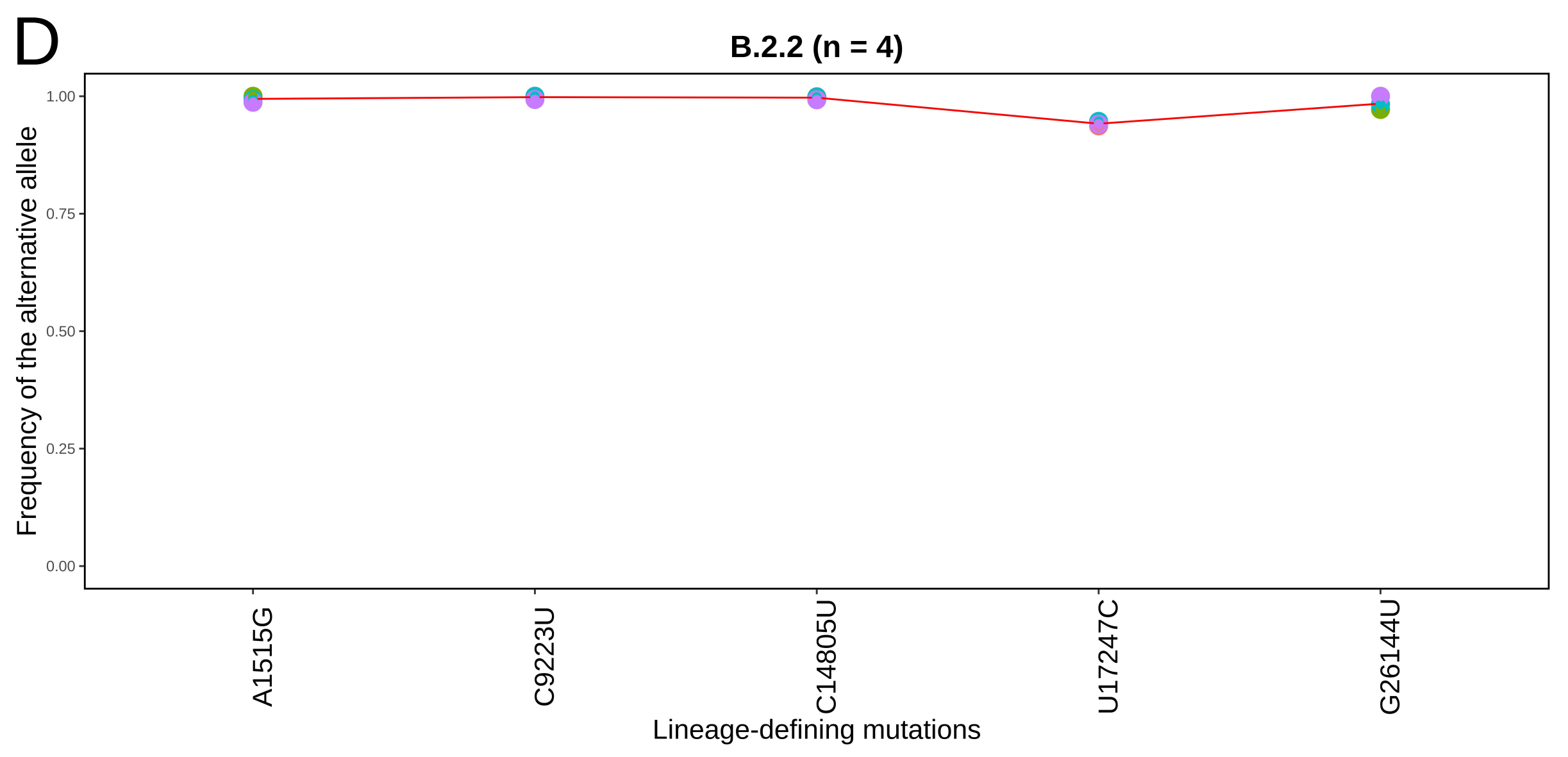

Supplement: veab078_Supp [file veab078_supp.zip › Figure_S4.pdf]

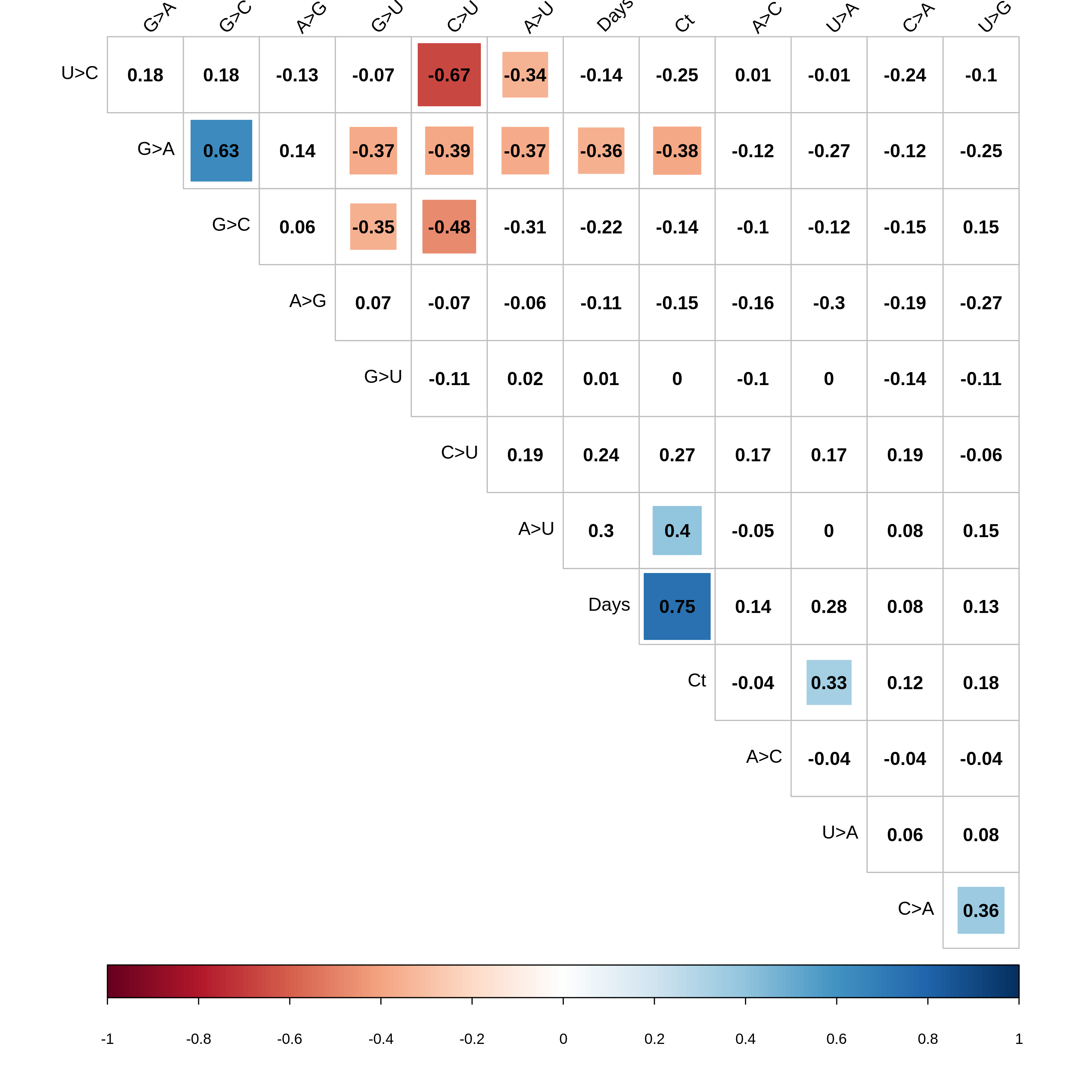

Supplement: veab078_Supp [file veab078_supp.zip › Figure_S6.png]
